# DNA secondary structures are associated with recombination in major *Plasmodium falciparum* variable surface antigen gene families

**DOI:** 10.1093/nar/gkt1174

**Published:** 2013-11-16

**Authors:** Adam F. Sander, Thomas Lavstsen, Thomas S. Rask, Michael Lisby, Ali Salanti, Sarah L. Fordyce, Jakob S. Jespersen, Richard Carter, Kirk W. Deitsch, Thor G. Theander, Anders Gorm Pedersen, David E. Arnot

**Affiliations:** ^1^Centre for Medical Parasitology, Department of International Health, Immunology and Microbiology, Faculty of Health and Medical Sciences, University of Copenhagen, Øster Farimagsgade 5, Building 22 & 23, PO Box 2099, 1014 Copenhagen K, Denmark, ^2^Centre for Medical Parasitology, Department of Infectious Diseases, Copenhagen University Hospital (Rigshospitalet), Copenhagen K, Denmark, ^3^Department of Systems Biology, Center for Biological Sequence Analysis, Technical University of Denmark, 2800 Lyngby, Denmark, ^4^Department of Biology, University of Copenhagen, Ole Maaloees Vej 5, DK-2200 Copenhagen N, Denmark, ^5^Centre for GeoGenetics, Natural History Museum of Denmark, University of Copenhagen, Øster Voldgade 5-7, 1350 Copenhagen, Denmark, ^6^Institute of Infection and Immunology Research, School of Biological Sciences, University of Edinburgh, West Mains Road, Edinburgh EH9 3JT, Scotland, UK and ^7^Department of Microbiology and Immunology, Weill Cornell Medical College, New York, NY 10065, USA

## Abstract

Many bacterial, viral and parasitic pathogens undergo antigenic variation to counter host immune defense mechanisms. In *Plasmodium falciparum*, the most lethal of human malaria parasites, switching of *var* gene expression results in alternating expression of the adhesion proteins of the *Plasmodium falciparum*-erythrocyte membrane protein 1 class on the infected erythrocyte surface. Recombination clearly generates *var* diversity, but the nature and control of the genetic exchanges involved remain unclear. By experimental and bioinformatic identification of recombination events and genome-wide recombination hotspots in *var* genes, we show that during the parasite’s sexual stages, ectopic recombination between isogenous *var* paralogs occurs near low folding free energy DNA 50-mers and that these sequences are heavily concentrated at the boundaries of regions encoding individual *Plasmodium falciparum*-erythrocyte membrane protein 1 structural domains. The recombinogenic potential of these 50-mers is not parasite-specific because these sequences also induce recombination when transferred to the yeast *Saccharomyces cerevisiae*. Genetic cross data suggest that DNA secondary structures (DSS) act as inducers of recombination during DNA replication in *P. falciparum* sexual stages, and that these DSS-regulated genetic exchanges generate functional and diverse *P. falciparum* adhesion antigens. DSS-induced recombination may represent a common mechanism for optimizing the evolvability of virulence gene families in pathogens.

## INTRODUCTION

The pathogenicity of *Plasmodium falciparum* malaria is linked to the parasite’s capacity to modify infected erythrocyte surfaces to cause sequestration in various host organs, thus allowing the parasites to avoid clearance by the spleen (1). Sequestration of infected erythrocytes is mediated by binding of parasite-encoded *Plasmodium falciparum*-erythrocyte membrane protein 1 (PfEMP1) adhesion antigens to host endothelial receptors (2). PfEMP1 are major, immunodominant targets of protective antibody mediated immunity (3). To escape antibody mediated immunity, which will tend to be antigen variant-specific, parasites have evolved to contain a repertoire of ∼60 *var* genes encoding different PfEMP1 variants (1). Inter-genome comparisons have revealed an immense variation in the global pool of *var* gene sequences (4,5). This opposing evolutionary pressure on PfEMP1 molecules to generate immune-evading antigenic diversity, while maintaining receptor binding capacity, has resulted in highly organized genetic structures harbouring alternating loci with relatively conserved and extremely diverse sequences. *Var* genes can be classified into three major groups (A–C) on the basis of their 5′ upstream sequences (*upsA, B and C)* and chromosomal location and orientation (6). The encoded PfEMP1 consist of restricted compositions of different subtypes of Duffy Binding Like (DBL) and cysteine-rich interdomain region (CIDR) domains, which are associated with the *ups* type of the genes (7), specific cytoadhesion properties (i.e. human receptor specificities (2) and, ultimately, disease outcome (8,9).

This genetic organization and functional compartmentalization is thought to be maintained not only through selection, but also as a consequence of a restricted recombination imposed by e.g. the different chromosomal positioning and orientation of the centromeric *ups*C *var* genes and the inversely oriented subtelomeric *ups*A and *ups*B genes (6,10). There is general consensus from experimental and sequence analysis studies that *var* genes are subject to ectopic recombination causing gene conversion (11–15). Studies on experimental genetic crosses have presented evidence that recombination between *var* genes on heterologous chromosomes occurs more frequently than expected from the overall estimated rate of meiotic crossing-over (12,14,16,17), and data from these and other studies indicate that chimeric *var* genes are products of recombination between isogenous *var* paralogs (i.e. *var* genes originating from the same haploid *P. falciparum* genome) (12,14,18,19). These data suggest that *var* recombination is preferentially initiated in the sexual stages, although *var* genes have also been documented to recombine during the mitotic divisions of asexual blood-stage parasites (18,20,21). However, as noted by Bopp *et al.* in 2013, it appears unlikely that *var* chimeras generated during the sexual reproduction and by mitotic recombination occurring in asexual blood-stages are products of the same recombination mechanism. It has been proposed that clustering of chromosome-ends into bouquet-like configurations, observed in the sexual parasite forms, facilitates genetic exchanges between subtelomeric *var* genes by securing the necessary proximity (12). The identification of inducing and regulating factors that can explain *how* and *when var* recombination is initiated is essential to understand how the parasite is able to employ a strategy of immune evasion through *var* gene diversification.

Here we document a tight association between predicted DNA secondary structures (DSS) and *var* gene recombination sites in four chimeric *var* genes, known recombination hotspots and boundaries of protein domains. Altogether these data indicate that DSS serve as *var* recombination inducers and suggest a hitherto unseen arrangement to facilitate ordered recombination.

## MATERIALS AND METHODS

### *P. falciparum* culture

*P. falciparum* blood-stage progeny clones (X2, X4, X6, X8, X10, X11, X12, X30, X44, X47, X50, X56 and X67) from the HB3x3D7 cross (16) were cultured as described (22).

### Pulsed field gel electrophoresis and quantitative real-time polymerase chain reaction

*P. falciparum* chromosomal DNA blocks were prepared as described (23). Pulsed field gel electrophoresis (PFGE) was performed as described (24). Separated chromosomes were excised from the PFGE gels and DNA purified using spin columns. Primers for 60 3D7 (25,26) and 49 HB3 (27) *var* genes, and 14 syntenic chromosome marker genes (Supplementary Table S1a), were used in quantitative real-time polymerase chain reaction (QPCR) to determine the chromosomes and *var* genes contained in each excision (28). QPCR primers (Supplementary Table S1b) targeting 5′and 3′ends of 49 3D7 and 29 HB3 *var* genes were applied to progeny genomic DNA to identify chimeric genes lacking either the 5′ or 3′ end of the original parental genes. Primer amplification efficiencies were validated by QPCR on serial dilutions of genomic DNA. QPCR was performed using the Rotorgene 6000, version 1.7 system (Corbett Research). Reactions were prepared in 20-µl aliquots using the QuantiTect SYBER Green PCR kit (Qiagen) and 1 µM primer concentrations. PCR cycling was 95°C for 15 min, followed by 40 cycles of 95°C for 30 s, 54°C for 20 s and 65°C for 40 s, with final extension at 68°C for 40 s. The cycle threshold was set at 0.025 and all products were authenticated by melting point analysis.

### Amplification of *var* exon 1 for sequencing and restriction fragment length polymorphism analysis

#### PCR amplification of var exon 1

*var* exon 1 sequences were amplified using primers listed in Supplementary Table S1c. PCR reactions were done using TaKaRa LA Taq™ polymerase (Fisher) following the manufacturer’s recommendations. Two-step PCR conditions were one cycle of 94°C for 1 min, followed by 33 cycles of 98°C for 10 s and an annealing/extension at 60°C for 5 min. The identity of individually amplified *var* exon 1 sequences was tested by QPCR using primers against all 3D7 *var* genes.

#### RFLP analysis

Seventy-one amplified *var* exon 1 from 3D7 and seven progeny clones were digested with five different restriction enzymes (Taq I, Eco RI, Hpa I, Hha I and Cla I) and visualized on agarose gels.

#### Sequencing

Fifty-one amplified *var* exon 1 sequences (size 5–10 Kb), together with chromosomes 2–4 of progeny X5, were sequenced on a Roche FLX platform using Titanium sequencing chemistry. Amplified *var* gene sequences from the same progeny clone were pooled in equimolar concentrations (20–50 ng/µl), and then fragmented using Fragmentase (New England Biolabs) to an average size of <1000 bp. DNA fragments were purified using a Minelute column (Qiagen), then build into MID labelled sequencing libraries according to the manufacturer’s guidelines and the Rapid FLX library build kit (Roche). Each library was subsequently subjected to emulsion PCR and sequenced. Post-sequencing data were firstly split by the multiplex identifier (MID) of each library, then de novo assembled by Newbler v2.3 (454 Life Sciences Corp., A Roche Company, Brandford, CT 06405, USA), and finally analysed using BioEdit.

### Identification and validation of X4 PFB0010w/PFA0765c chimera

Using QPCR and primer pairs specific for 5′ and 3′ ends of 78 parental *var* genes, the 5′ but not the 3′end of 3D7 chromosome 2 gene PFB0010w and the 3′ but not the 5′ end of 3D7 chromosome 1 gene PFA0765c could be amplified from genomic DNA of progeny clone X4. To test if the two parental genes, PFB0010w and PFA0765c, had recombined to create a chimeric gene in the X4 progeny clone, the PFB0010w 5′-end forward primer and the PFA0765c 3′-end reverse primer were subsequently used to amplify the possible chimeric sequence. These primers successfully amplified a 5500-bp sequence from genomic DNA of progeny X4, but failed to amplify any product from 3D7 genomic DNA. Sequencing of the 5500-bp PCR product showed a chimeric sequence containing the 5′-end of PFB0010w and the 3′-end of PFA0765c. The gene-specific QPCR primer pairs specific for the 5′ and 3′-end of the X4 PFB0010w/PFA0765c chimera were then tested on PFGE-separated chromosomal DNA from progeny clone X4 together with chromosome marker primers for each of 14 chromosomes. This analysis showed that both ends of the chimeric X4 PFB0010w/PFA0765c gene were contained within the chromosome block containing chromosomes 2–4, and thus confirmed that ectopic recombination had resulted in translocation of the PFA0765 3′-end from chromosome 1 to chromosomes 2–4. To exclude the possibility that PCR amplification had produced an artefact chimeric sequence, we designed PCR primer pairs based on the X4 PFB0010w/PFA0765c chimeric sequence that specifically should amplify regions containing the observed recombination breakpoints. PCR amplification using these primer pairs failed to amplify any products from 3D7 genomic DNA, but successfully amplified sequences of the right sizes from genomic DNA of the X4 progeny clone. Sequencing of these PCR products confirmed the presence of each of the sequences containing the observed recombination breakpoints in the X4 PFB0010w/PFA0765c chimeric sequence. Finally, we were able to extract single sequence reads from whole-genome sequencing data of 3D7 × HB3 progeny (available from Sanger Institute Malaria Program).

### Identification and validation of X5 PFA0005w/PFB1055c chimera

Gene mapping of inherited *var* genes in recombinant progeny clones showed that a central region of the PFA0005w gene located on chromosome 1 in 3D7 mapped to chromosomes 2–4 in progeny clone X5. This indicated that ectopic recombination had resulted in translocation of this PFA0005w-specific sequence from chromosome 1 to chromosomes 2–4. A primer pair specific for the 3′-end of the PFA0005w gene failed to amplify any sequence from genomic DNA of the X5 progeny clone. A forward primer specific for the 5′-end of the PFA0005 gene was then used together with a reverse primer targeting a conserved 3′ end sequence contained by most *var* genes to amplify the possible chimeric sequence in the X5 progeny. PCR amplification resulted in a ∼5500-bp product, which then was sequenced. The sequenced PCR product showed a chimeric sequence containing the 5′-end of the PFA0005w gene and the 3′-end of the PFB1055c gene located on chromosome 2 in 3D7. To ensure that this sequence did not result from a PCR artefact, we designed primers specific for each of the regions covering the observed recombination breakpoints. PCR amplification using these primer pairs failed to amplify any products from 3D7 genomic DNA, but successfully amplified sequences of the right sizes from genomic DNA of the X5 progeny clone. Sequencing of these PCR products confirmed the presence of each of the sequences containing the observed recombination breakpoints in the X5 PFA0005w/PFB1055c chimeric sequence. Finally, we were able to extract single sequence reads from whole-genome sequencing data of 3D7 × HB3 progeny (available from Sanger Institute Malaria Program and from shotgun sequencing of chromosome 2–4 of X5) covering each of the breakpoints.

### Identification and validation of X96 DD2var18/DD2var23 and X98 HB3var10/HB3var14 chimeras

Using BWA mapping software (version 1.2.2) (29) available on the public Galaxy server (usegalaxy.org) (30) Illumina GA II, 75-bp paired-end sequencing reads with at least one read matching one of the published DD2 and HB3 (31) *var* gene sequences were extracted from whole-genome sequencing data from 18 DD2 × HB3 progeny available from Sanger Institute Malaria Program (ENA accession number ERP000199). Paired-end reads from each progeny were *de novo* assembled separately using SOAPdenovo (32) (settings: k-mer size of 57 bp and median insert size as determined from the mapping) and compared with parental genes by alignment. This resulted in discovery of two contigs representing chimeric genes X96 DD2var18/DD2var23 (ERS009996) and X98 HB3var10/HB3var14 (ERS009998). Each breakpoint was validated by identification of paired reads, where one read contained the breakpoint and the mate read matched one of the parental genes.

### Bioinformatics

#### DNA 50-mer folding free energy calculations

All gene groups (*var* genes from six genomes as previously described (31), and all 3D7 CDS divided into *var*, *rif*, *stevor* and other genes) were subjected to a moving window analysis, using a window size of 50 nt and step size of 1 nt. The minimum free energy structure was calculated for all 50-mers using RNAfold v2.1.2 with thermodynamic parameters specifically for folding single-stranded DNA sequences (parameter file dna_mathews1999.par from the ViennaRNA package v2.1.2) (33). GU and lonely pairs were disallowed in the secondary structures. Graphical models of shown 50-mer DSS were drawn using Context fold (34).

Shuffled versions of the gene groups were also analysed as described earlier. The shuffled gene groups were generated by randomizing the nucleotide sequence of each gene, thus producing sequence sets with same gene number, length and nucleotide composition. Density and frequency plots were produced using scripts of Python v2.5 and R v2.8. Plots and statistical tests were generated for the 1%, 2%, 3%, 4% and 5% of the lowest 50-mer folding free energies calculated in the shuffled *var* genes, to define an energy threshold of 50-mer sequences predicted to form DSS. Calculations based on all five thresholds gave similar results and conclusions, but only the results of the 3rd percentile data (corresponding to an energy threshold of −6.27 kcal/mol) are presented.

### Statistical analysis of correlation between recombination sites and predicted DSS

Statistical significance of the correlation between recombination sites and predicted DSS in the four chimeric genes was tested using re-sampling based analysis. The null hypothesis was that recombination sites occurred at random distances from DSS. First the average distance between recombination sites and the nearest DSS in either of the two parental donor sequences was calculated (average distance was 44.46 nucleotides). This value was compared with the corresponding distribution of average distances between randomly chosen locations in the four sets of parental genes and the nearest DSS. Specifically, the procedure was as follows: (i) One random location was selected in each of the two parental genes for a given chimera. (ii) The nearest DSS for this pair of locations was found (i.e. the DSS closest to either of the two random locations in the two parental genes). (iii) Steps 1 + 2 were repeated 13 times over the four parental gene pairs, such that the pairs were sampled the same number of times as for the actual observed chimeric genes (i.e. for each random average, we sampled three random distances from PFB0010w + PFA0765c, four from HB3var10 + HB3var14, one from DD2var23 + DD2var18 and five from PFB1055c + PFA0005w). 

(iv) The average of these 13 random DSS-distances was computed. (v) Steps 1–4 were repeated 1 000 000 times. (vi) Finally, the real average distance (44.46) was compared with the resulting distribution of 1 000 000 random average distances. Among these values, 9032 were found to be less than or equal to the real value. The probability of observing a value less than or equal to the real average distance if the null hypothesis is true (the *P*-value) is therefore 9032/1 000 000 = 0.009032.

### Statistical analysis of correlation between predicted DSS and previously defined recombination hotspots

Re-sampling based statistical analysis was performed on each PfEMP1 DBL domain type to test whether the number of DBL sequences containing a DSS very near to the previously defined recombination hotspot, located between the structural sub-domains S2 and S3, was significantly higher than expected for random reasons. Specifically, for each DBL domain type, the number of genes that contained one or more DSS within a window of ±50 nucleotides, surrounding the defined recombination hotspot, was counted. The same analysis was subsequently repeated on 1 000 000 shuffled datasets, in which the position of DSS found in each gene was randomly placed. The number of DSS in the real dataset was then finally compared with the number of DSS in the shuffled dataset to calculate the *P*-value. A similar procedure was performed within a window of ±50 nucleotides around the other previously defined recombination hotspot (31,35) at the ‘mid-*var* region’ of PfEMP1 type 1 genes (36) (specifically defined as the point halfway between the 3′-end of the NTS-DBLα-CIDR1 domains and the 5′-end of DBLδ-CIDR2 domains).

### Construction of yeast recombination strains

To insert a DSS sequence into a previously described direct-repeat recombination assay strain ML144-8C (37), the *KANMX4* selection marker was amplified from yeast genomic DNA (38) using homology and DSS-adapted primers LEU2-U2-KanMX-F (5′-GGATATCGTCCATTCCGACAGCATCGCCAGTCACTATGGCGTGCTGCTAG **CGTACGCTGCAGGTCGAC**) and PFB1055c-D2-KanMX-R (5′-*ACTTTTGCCAGTGGCACCA***ATCGATGAATTCGAGCTCG**), PFB1055cS-D2-KanMX-R (5′-*CCTAGCCAGCAATTTCAGG***ATCGATGAATTCGAGCTCG**), PFB1055bc-D2-KanMX-R (5′-*TGCCGTTTTCGTCCTTAC***ATCGATGAATTCGAGCTCG**), PFB1055bcS-D2-KanMX-R (5′-*TGTTGGTTTCCAGGCTTATAC***ATCGATGAATTCGAGCTCG**), PFA0765c-D2-KanMX-R (5′-*GGTTTTCTCACCAGGTGTTTG***ATCGATGAATTCGAGCTCG**) or PFA0765cS-D2-KanMX-R (5′-*GGCTTCCTTGCATATCAGTGC***ATCGATGAATTCGAGCTCG**). *KANMX4* marker homology sequence is shown in bold and DSS in italic. The two PCR products were subsequently extended with the full DSS sequence and 50 bp of homology (underscored) to the yeast genomic region of the direct-repeat recombination assay using primers PFB1055c-LEU2-R (5′-ACTATTTCTCATCATTTGCGTCATCTTCTAACACCGTATATGATAATATA*TGGGTGGCACACAAATGGCACCCTTATCACCACTTTTGCCAGTGGCACCA*), PFB1055cS-LEU2-R (5′-ACTATTTCTCATCATTTGCGTCATCTTCTAACACCGTATATGATAATATA*TTATAGGTCTACCCCGGGCACATCTAGGACCCCTAGCCAGCAATTTCAGG*), PFB1055bc-LEU2-R (5′-ACTATTTCTCATCATTTGCGTCATCTTCTAACACCGTATATGATAATATA*GGGGACTTGGTCGGCATTTGAGCCGGGCTTTTTGCCGTTTTCGTCCTTAC*), PFB1055bcS-LEU2-R (5′-ACTATTTCTCATCATTTGCGTCATCTTCTAACACCGTATATGATAATATA*CGCTTGGTAGGCGGGTTTCGGCCTTTCGCTGTTGGTTTCCAGGCTTATAC*), PFA0765c-LEU2-R (5′-ACTATTTCTCATCATTTGCGTCATCTTCTAACACCGTATATGATAATATA*GCACCCTGGTTAGTACCACTAGGTGGGGTGGTTTTCTCACCAGGTGTTTG*) or PFA0765cS-LEU2-R (5′-ACTATTTCTCATCATTTGCGTCATCTTCTAACACCGTATATGATAATATA*AGAGGCTTCGCGTCTTTGAGGATTGCTGGGGCTTCCTTGCATATCAGTGC*) together with primer LEU2-U2-KanMX-F. The two fusion PCR products were separately transformed into yeast strain ML144-8C using the lithium acetate method (39). Transformants were selected on YPD containing 200 µg/ml G418 (Sigma-Aldrich). Correct integration of the PCR products was confirmed by sequencing.

#### Recombination assay

Direct-repeat mitotic recombination was measured in haploid yeast strains. The procedure for determining mitotic recombination frequencies and their standard deviation was essentially as described before (40), with the following exception: All cultures were grown in liquid synthetic complete medium supplemented with 100 µg/ml adenine (41). Single-strand annealing was distinguished from gene conversion by replica-plating the Leu^+^ recombinants to synthetic complete medium lacking leucine and uracil after 2 days to score for loss of the *URA3* marker. The median frequency for 15 trials was used to determine the recombination rate by the method of the median (41).

## RESULTS

### Identification of chimeric *var* genes

To study *var* gene recombination events, progeny clones of a genetic cross (16) between two *P. falciparum* clones, 3D7 and HB3, were searched for novel chimeric *var* genes using three complementary approaches. *Var* genes consist of a large 3–10-kb polymorphic exon 1 encoding the variable extracellular domains and a conserved 1.3-kb exon 2 encoding the intracellular domain. Fourteen of the cross progeny were analysed using QPCR and primer pairs specific for the 5′ and 3′ ends of exon 1 of 78 parental *var* genes (this constitutes 72% of all the parental *vars*). To detect ectopic recombination events between non-allelic *var* genes, the chromosomal localization of all parental *var* genes was also mapped in both parents and seven of the progeny clones. This was carried out using *var* gene and chromosome-specific marker primers in QPCR screens of PFGE-separated chromosomes (28), and the experimental results were subsequently compared against the complete 3D7 genome data available from the PlasmoDB database (http://plasmodb.org/plasmo/) (Supplementary Figure S1). Finally, 120 full-length randomly chosen *var* exon 1 sequences were PCR amplified and either sequenced (n = 49) or compared for RFLPs (n = 71). In addition to these experiments, whole-genome sequencing data from 18 progeny clones of a second cross (clone HB3 × DD2), available from Sanger Institute, were also searched in an effort to identify other recombinant *var* chimeras.

Four recombinant *var* genes were identified in these screens. In two of the 3D7 × HB3 progeny clones, the QPCR analysis indicated that ectopic recombination had occurred between the 3D7 chromosome 1 and 2 *var* genes. The novel chimeric genes were located on chromosome 1 in progeny clone X4 and chromosome 2 in progeny clone X5. Additional PCR analyses and shotgun sequencing of clone X5 chromosomes 2–4 (not shown) confirmed that both recombinant genes (X5_PFA0005w/PFB1055c_ and X4_PFA0765c/PFB0010w_) were chimeras resulting from an ectopic recombination between two *upsB var* genes that had the same orientation and telomeric position, although they were situated on different chromosomes of the 3D7 parent.

PCR experiments confirmed both presence of the two donor genes and the absence of the chimeric genes found in the progeny, in the parental 3D7 genome. Chromosome mapping of *var* genes in progeny clone X5 showed that this parasite genome only contains HB3 chromosome 1 *var* genes on its chromosome 1 (Supplementary Figure S1). This indicates that the process of meiotic crossing-over has replaced 3D7 chromosome 1 loci, originally containing the recombining PFA0005w gene, with HB3 DNA after the recombination event. Together with PCR experiments, confirming both the presence of donor genes and the absence of chimeric progeny genes in the parental 3D7 genome, this indicates that the isogenous *var* recombination creating X5_PFA0005w/PFB1055c_ occurred sometime between the onset of gametogenesis and the meiotic reduction divisions in the ookinetes in the mosquito midgut.

**Figure 1.**
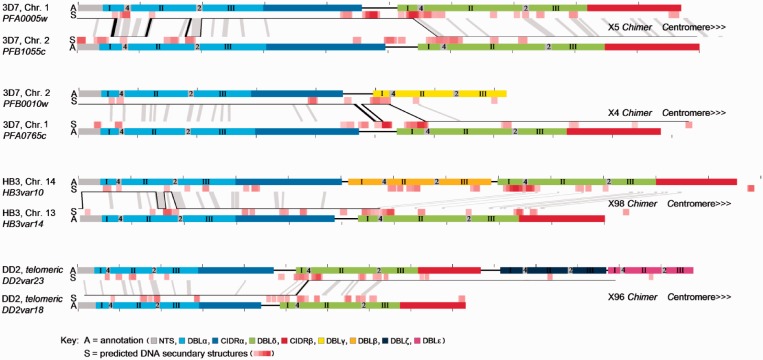
Schematic diagram of the origin of four novel *var* chimeric genes. The verified sequences from four chimeric *var* genes (exon 1) from two 3D7 × HB3 progeny (X5 and X4) and two HB3 × DD2 progeny (X98 and X96) are shown as continuous black lines crossing between domain-annotated parental genes. These are all *upsB* type *var* genes, i.e. telomeric genes being transcribed towards the centromere. PfEMP1 are composed of the N-terminal segment (NTS) and different sub-classes of DBL and CIDR domains (coloured). DBL domains contain three structural elements, sub-domains 1–3 (marked I–III) (42,43). Both DBL and CIDR domains can be described as being composites selected from a repertoire of 628 different short semi-conserved homology blocks (HB) (31). Six breakpoints occurred at the boundaries of the structural DBL sub-domains 1–3 marked by HB4 and HB2 (grey boxes ‘2’ and ‘4’) and two breakpoints occurred in low-complexity sequence inter-domain regions. The quasi-palindromes with highest potential to form DSS shown in red (S) are frequently found near recombination breakpoints. Predicted DSS are identified by calculations of folding free energy in 50-mer windows (Supplementary Figure S2) and coloured increasingly intense red with decreasing folding free energy levels from −6.27 kcal/mol. Regions of donor sequence identity of at least 90% over 20 bp are shown in grey shades between genes. 500-bp intervals are marked.

Two additional chimeric *var* sequences (X96_DD2var18/DD2var23_ and X98_HB3var10/HB3var14_) also generated by isogenous recombination between *upsB var* genes were identified in the analysis of the whole-genome sequencing data resulting from the other genetic cross between parental clones HB3 and DD2. A schematic diagram of the sequenced chimeric *var* genes showing the recombination breakpoints relative to the parent donor genes is shown in Figure 1 and in high detail in Supplementary Figure S2.

### Recombination breakpoint analysis

The recombination patterns of the chimeric genes are similar and give a novel insight into the mechanism of *var* recombination. All four sequences have between one and five recombination breakpoints and retain open reading frames (Supplementary Figure S2), indicating that they could be expressed as functional proteins. Interestingly, 8 of the 13 breakpoints are located at or close to the boundaries of sequences encoding distinct PfEMP1 tertiary structures (Figure 1) i.e. six recombination breakpoints are located at the boundaries of the three structural sub-domains that make up the conserved fold of DBL domains (42,43), whereas two breakpoints are located in the low-complexity inter-domain region of X4_PFA0765c/PFB0010w_. Both the region separating DBL sub-domains 2 and 3 and the low-complexity inter-domain region have previously been associated with high frequencies of recombination (31). This indicates that these regions are recombination hotspots *per se* (i.e. recombination occurs more frequently within these defined regions than elsewhere in the genes) and that recombination is being actively directed to these regions.

The sequences around each breakpoint were closely examined for features possibly relating to their recombinogenic propensity. All breakpoints occurred within highly similar regions between the parental donor sequences, with a minimal required sequence identity of around 20 base pairs with 10% mismatch. Interestingly, numerous short quasi-palindromes (i.e. imperfect inverted repeats separated by very few base pairs) were also observed located near the recombination breakpoints (Supplementary Figure S2). The recombinogenic potential of secondary hairpin or cruciform DNA structures formed by quasi-palindromes has recently attracted considerable interest, with several studies presenting examples of individual recombination sites being marked by the presence of a palindromic DNA sequence [reviewed in (44,45)]. Mitotic recombination analysis in *Saccharomyces cerevisiae* indicates that recombination breakpoints occur at distances up to several kilobases from an initiating DSS. The highest probability of recombination being in homologous regions closest to the DNA secondary structure (46). This is comparable with the recombination events observed here. If DSS are a primary initiator of *var* recombination, during the course of evolution, these structures should become concentrated at or near recombination hotspots. To test this hypothesis, we investigated the location of predicted DSS in the donor sequences of the *var* chimeras as well as within a large sample of sequenced *var* genes.

### DSS locations are associated with *var* recombination hotspots and PfEMP1 domain borders

The folding free energy of single-stranded DNA was calculated in a sliding 50-mer window across all *P. falciparum* genes. The *var* genes were found to harbour a high proportion of particularly low folding free energy 50-mers with the highest likelihood of forming DSS (Supplementary Figure S3 and examples of 50-mer DSS given in Supplementary Figure S4). To visualize the positioning of these sites with the potential to form DSS, the lowest folding free energy DSS were plotted onto the parental donor sequences of the identified *var* chimeras (Figure 1) and onto 366 full-length domain-aligned PfEMP1 sequences, following a recently published general PfEMP1 domain annotation scheme (31) (Supplementary Figure S5). Plots were generated using energy thresholds corresponding to 1st–5th percentiles of 50-mers in randomized *var* gene sequences. All plots exhibited similar DSS localization patterns, but only the 3rd percentile data are shown here (folding free energies below −6.27 kcal/mol).

Visual inspection of the four *var* chimeras indicated a correlation between the identified recombination sites and locations of low folding free energy 50-mers (predicted DSS) in the corresponding parental donor sequences. To investigate this correlation statistically, we performed a re-sampling based analysis (see ‘Materials and Methods’ section), which showed that the average distance between a recombination break site and the nearest corresponding DSS (in either of the donor sequences) was significantly lower than that expected for random associations (*P* = 0.009032).

**Figure 2.**
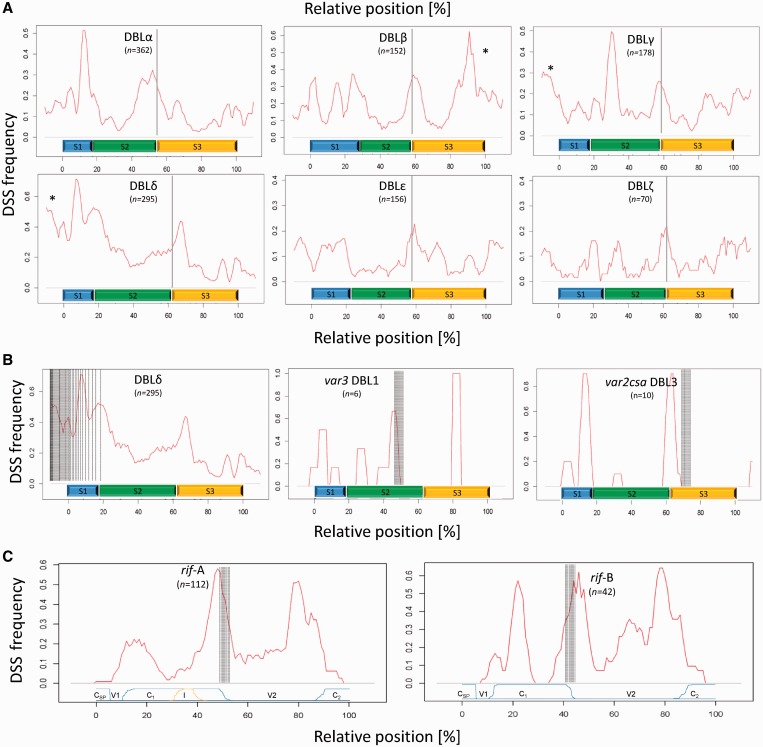
Relative location of DSS in selected *var* gene domains and *rif* genes. (**A**) The frequency of predicted DSS (red graph) is shown relative to the position in the PfEMP1 domain types DBLα–ζ. The relative position of DBL sub-domains S1–S3 (blue, green and orange bars) and the previously defined recombination hotspot in DBL domains (vertical grey lines) (31) is shown. In general, peaks in DSS frequency are observed at the recombination hotspot located at the boundary of DBL S2 and S3 and at the end of the domains (indicated by asterisk). The frequency of DSS at the DBL S2–S3 recombination hotspot was found to be significantly higher than expected by chance (Table 1). (**B**) The association between DSS localization and the ‘mid var’ recombination region (marked by vertical punctuated lines) is particularly evident in the DSS frequency plot of DBLδ domains (left plot). The recombination hotspots (vertical grey lines) defined in *var3* DBL1 sub-domain 2 (31) and *var2csa* DBL3 (47) also co-localize with peaks in DSS frequency (middle and right plots). (**C**) The frequency of predicted DSS (red graphs) is shown relative to their position in annotated *rif*-A and B genes. Blue line indicates the relative positions of conserved (Csp = conserved signal peptide, C1 and C2) and variable (V1 and V2) regions. Yellow line indicates position of the 75-bp insert (I) unique to *rif*-A. In both *rif*-A and B genes, the highest DSS frequency peaks are found at the border between major conserved (C1) and variable (V2) regions (grey shadow), previously defined as a hotspot for recombination (48).

Mapping of low folding free energy 50-mers in 366 domain-aligned PfEMP1 sequences (Supplementary Figure S5) showed that predicted DSS appeared to be non-randomly localized, with a striking tendency to occur at recombination hotspots previously proposed by studies of sequence homology disequilibria within the PfEMP1 family. To investigate this in detail, the frequency of DSS occurrence was plotted along the sequence of each of the main DBL and CIDR domain types (Figure 2 and Supplementary Figure S6). The high frequency of predicted DSS in and around the 5′ end of DBLδ domains is particularly evident. This ‘mid-*var* gene region’ has been reported to be a site of frequent recombination between the so-called type 1 *var* paralogs (i.e. DBLα-CIDR1- DBLδ -CIDR2; Supplementary Figure S5) (31,35). A re-sampling based statistical analysis confirmed that the number of *var* genes containing a DSS within a window of ±50 nucleotides around this ‘mid-*var* gene region’ (defined as the point halfway between the 3′ end of the CIDR1 domain and the 5′ end of the DBLδ domain of type 1 *var* genes) was significantly higher than the random expectation (Table 1). Sequence analysis of DBL sub-domains 1–3 has also identified the border between DBL sub-domains 2 and 3 as a hotspot for recombination (31). Figure 2 shows a high frequency of predicted DSS at the borders of DBL sub-domains 2–3, which also were tested to be significantly higher than expected by chance (Table 1). The DSS frequencies at previously *in silico* defined recombination hotspots in the conserved *var2csa* (47) and *var3* genes (31) were also investigated (Figure 2b) and again found to be associated with high frequencies of DSS.

**Table 1.** Test results of correlation between predicted DSS and previously defined recombination hotspots

Hotspot	Number of sequences	Number of sequences with DSS within ±50 bp of hotspot		
		Real	Random [CI]	*P*
‘Mid var’	146	112	36 [15, 66]	<0.000001
DBLα S2–S3	362	136	85 [48, 124]	<0.000001
DBLβ S2–S3	152	85	34 [12, 63]	<0.000001
DBLγ S2–S3	178	59	37 [15, 64]	0.000109
DBLδ S2–S3	295	189	71 [35, 109]	<0.000001
DBLε S2–S3	156	45	28 [9, 54]	0.000690
DBLζ S2–S3	70	21	13 [0, 31]	0.014570

### DSS locations are associated with *rif* gene recombination hotspots and RIFIN domain borders

The *rif* genes constitute *P. falciparum’*s largest variant surface antigen family with ∼150 gene copies/genome (36). These genes also contain a high density of low folding free energy 50-mers, relative to other *P. falciparum* genes (Supplementary Figure S3). The fact that *rif* and *var* genes share both hyper-variation and frequent telomeric location suggests that similar recombination mechanisms may operate on both gene families (11,48). RIFINs can be divided into A and B types, based on a 75-bp insert specific to RIFIN-A types. Recombination is expected to occur most frequently at the border between the major conserved (C1) and variable (V2) domain (48), coinciding with the recombination breakpoint in the only known *rif* chimera (11,48). Figure 2c shows that predicted DSS are concentrated around the expected recombination hotspot, supporting a hypothesis that *rif* and *var* genes evolve via similar DSS-induced recombination.

### *Var* 50-mers predicted to form DSS induce recombination in yeast

**Figure 3.**
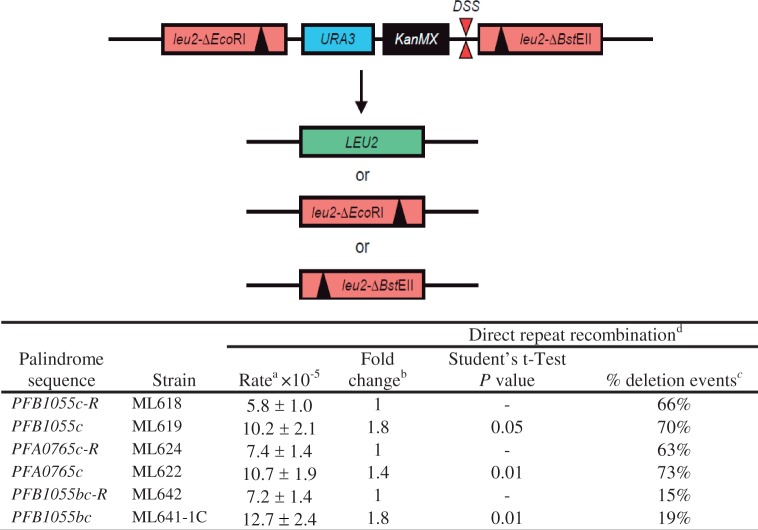
Effects of predicted DNA secondary structure sequences on mitotic leu2 direct-repeat recombination. The schematic illustrates the assay for spontaneous direct-repeat recombination between two non-functional leu2-ΔEcoRI and leu2-ΔBstEII alleles showing the position of the inserted DSS sequences (DSS). Recombination between the leu2 alleles to produce a functional LEU2 allele leads to prototrophy for leucine (Leu+). The assay scores for Leu+ recombinants generated by single-strand annealing, replication slippage or gene conversion between the leu2 alleles. Single-strand annealing leads to loss of the URA3 gene and uracil autotrophy. The fraction of URA3 deletion events were the same for the pair-wise combinations of strains and their scrambled counterpart, indicating that the 50-mer with the lowest folding free energy stimulated the different types of recombination equally well. ^a^Recombination rate is presented as events per cell per generation ± standard deviation, as described in ‘Materials and Methods’ section. ^b^Relative to a randomized sequence (R). ^c^Percentage of deletion events among Leu^+^ recombinants. ^d^Strains harbouring the palindrome sequences PFB1055c (ML619: 5′-TGGTGCCACTGGCAAAAGTGGTGATAAGGGTGCCATTTGTGTGCCACCCA), PFA0765c (ML622: 5′-CAAACACCTGGTGAGAAAACCACCCCACCTAGTGGTACTAACCAGGGTGC) and PFB1055bc (ML641-1C: 5′-GTAAGGACGAAAACGGCAAAAAGCCCGGCTCAAATGCCGACCAAGTCCCC) or their randomized counterparts PFB1055c-R (ML618: 5′-CCTGAAATTGCTGGCTAGGGGTCCTAGATGTGCCCGGGGTAGACCTATAA), PFA0765c-R (ML624: 5′-GCACTGATATGCAAGGAAGCCCCAGCAATCCTCAAAGACGCGAAGCCTCT) and PFB1055bc-R (ML642: 5′-GTATAAGCCTGGAAACCAACAGCGAAAGGCCGAAACCCGCCTACCAAGCG), respectively, were analysed for mitotic direct-repeat recombination at 30°C, as described (49).

To assess the recombinogenic potential of specific 50-mers associated with actual recombination breakpoints in the X5_PFA0005w/PFB1055c_ and X4 _PFA0005c/PFB0010w_ chimeras, the PFB1055c_2886__–2935_, PFB1055_884-933_ and PFA0765_2863__–2912_ 50-mers and their randomized counterparts (of higher folding free energy) were cloned into the yeast *S. cerevisiae* and tested in a recombination assay commonly used to study homologous recombination (49). All the *var* 50-mers exhibited a significantly higher recombination rate (1.4–1.8-fold) compared with the randomized versions of these sequences (Figure 3).

## DISCUSSION

Genetic recombination requires that participating donor sequences have sufficient sequence identity, that the chromosomes involved in the event are in proximity and that some distinct factor initiates the process (50). While physical clustering of heterologous chromosome-ends may result in the necessary proximity (12), quasi-palindromic sequences prone to forming base-paired DSS appear to act as the inducing factors initiating recombination in *var* genes. DSS are known to induce recombination in other organisms by making the DNA more accessible to structure-specific nucleases (51), by replication fork stalling and collapse followed by micro-homology-mediated strand invasion (52,53) or by strand invasion-independent faulty template switching of the DNA polymerase during DNA replication (54). The proximity of multiple recombination breakpoints within the *var* chimeras identified here, which are the result of recombination between unlinked *var* paralogs, is most consistent with a template switching mechanism. Such template switching has been previously observed when the DNA replication fork has stalled (55).

Our screens to identify novel chimeric *var* genes in genetic cross-progeny clones yielded four chimeras all created by ectopic recombination between *var* genes of the same genome. This result proves the previously raised hypothesis that *var* recombination mainly occurs between isogenous *var* paralogs during sexual reproduction (12) and is also suggestive of a replication-dependent mechanism. Replication-dependent recombination may not be restricted to the sexual stages but could, in theory, also operate during the mitotic divisions of blood-stage parasites. However, the differences between the recombination events creating the chimeric *var* sequences identified here and the *var* chimeras created from mitotic recombination during culturing of asexual blood-stages (20) suggest that different responsible mechanisms have been in play. Specifically, whereas the chimeras created in asexual blood-stage parasites are generated by single crossing-over events with no association to DSS or PfEMP1 domain structures, the chimeras generated during sexual reproduction contain multiple closely located recombination breaks resulting in a mosaic gene composition characteristic of *var* genes (13,15).

The observation that subtelomeric regions of heterologous chromosomes associate differently in asexual and sexual parasite forms (in clusters near the nuclear periphery and in bouquet-like configurations near one pole of the elongated nuclei, respectively) (12) may have influence on the ability of *var* genes to recombine at the different time points. In addition, given the likely association with DNA replication, a stage-specific *var* recombination mechanism could be associated with the DNA replication phase when the chromosome complement doubles from 2N to 4N in the short-lived zygotic stage known as the ookinete (56) or with the three rapid genome doublings, which create the ‘male’ microgametes, although previous studies have reported that the Dd2 clone is unable to produce male gametocytes, making the latter notion less likely (57,58).

In consensus, our evidence supports the view that virulence gene diversification in *P. falciparum* results from ectopic recombination between isogenous paralogs caused by a DSS-induced mechanism during DNA replication and explains previous observations that the *P. falciparum* parasite is able to generate new *var* genes during self-mating of male and female gametes derived from a single clone (18). This ability has probably been advantageous to the parasite, as it enables diversification of progeny antigen repertoires despite high inbreeding rates, which have been measured at 0.34 under high-intensity transmission in Tanzania (59) and at 0.90 under lower-intensity transmission in Papua New Guinea (60).

PfEMP1 proteins can be understood as composites of partially conserved homology blocks, resulting from shuffling *var* gene segments under the constraint of maintaining functional cytoadhesive structures while modulating antigenicity in the attempt to evade variant-specific immunity. The finding that the *var* DNA with the highest likelihood of forming DSS co-localize with recombination hotspots at, or close to, breaks of homology and boundaries of structural elements in the conserved superstructure of PfEMP1/*var* genes is significant. It indicates that DNA structural features, and not just the protein phenotype, have been selected to increase the frequency of recombination at positions that optimize the chances that an antigenically novel PfEMP1 structure retains essential functional domains. These data are the first evidence of a DSS-dependent recombination mechanism regulating and directing the evolution of a gene family. The structural boundary ‘rules’ being followed may aid the definition and re-engineering of minimal binding regions of complex PfEMP1 adhesins, in the ongoing effort to develop PfEMP1-based vaccines and cytoadhesion-blocking anti-malaria therapy.

The DSS-directed mechanism of *P. falciparum* virulence gene recombination seems to have evolved under host selection, to optimize conservation of essential protein functions while generating sufficient antigenic diversity to escape preexisting immunity. The *var* DSS 50-mers were shown to induce recombination when tested in a *S. cerevisiae* recombination assay, indicating that the *var* DSS have an intrinsic recombinogenic potential, but it does not exclude that protein factors specific to *P. falciparum* may also contribute to promoting recombination at DSS sequences. The relatively low but nonetheless significant recombinogenic potential of individual *var* DSS 50-mers may reflect the constraint of allowing DNA replication to occur with reasonable fidelity while at the same time stimulating above background levels of recombination.

A recent study of African trypanosome species, the first identified and best understood model of protozoan antigenic variation, has shown that DNA helix-destabilizing TAA:TTA repeats within the VSG antigen genes induce antigenic variation and VSG sequence diversification through a recombination pathway that shares some resemblance to the mechanism of *var* gene diversification outlined here (61). Both the VSG and *var* multi-gene families evolve by ectopic recombination events through a mechanism of break-induced replication (62,63), but the inducing DNA structures appear to be different in the two parasites. Where specific TAA:TTA repeats destabilize the DNA helix of trypanosome VSG genes (64), *Plasmodium var* gene DSS are formed from diverse sequences and are predicted to form stable secondary structures. These differences may reflect the fundamentally different functional constraints on PfEMP1 (receptor binding) versus VSG (antigenic variation, ‘smoke screen’), as well as the fact that VSG gene expression frequently is switched by recombination (often of gene fragments from a large repertoire of pseuodogenes) into an active expression site (65), whereas switching of *var* gene expression appears to be independent of recombination (66) and involves *in situ* activation of intact genes, thus reducing the importance of pseudogenes. Various DNA structure-induced recombination pathways may thus have evolved in pathogens, each balancing immune selection pressure to create novel antigenic variants with disease-specific functional constraints.

## SUPPLEMENTARY DATA

Supplementary Data are available at NAR Online.

## FUNDING

The Danish National Research Foundation (http://www.dg.dk/) through a Niels Bohr Visiting Professorship award [Project: 312000-50-64920 to A.F.S. and D.E.A.]; Funded by Carlsbergfondet [2012_01_0393 to A.F.S.]; The Danish Council for Independent Research (to T.L.); The [FP7/2007-2013] under grant agreement [#200889 (STOPPAM) to A.S.]; the European Research Council (to M.L.); The Danish National Research Foundation and Lundbeck Foundation (to S.L.F.); a grant from the Danish Center for Scientific Computing (DCSC) (to T.S.R. and A.G.P.). Funding for open-access charge: Centre for Medical Parasitology, ISIM, University of Copenhagen.

*Conflict of interest statement.* None declared.

## Supplementary Material

Supplementary Data
