# Supplementary material for: DNA secondary structures are associated with recombination in major Plasmodium falciparum variable surface antigen gene families
Source: Nucleic Acids Res. 2013 Nov 16;42(4):2270–81. doi: 10.1093/nar/gkt1174 (PMC3936766; doi:10.1093/nar/gkt1174)
Supplement: Supplementary Data [file supp_42_4_2270__index.html]

DNA secondary structures are associated with recombination in major Plasmodium falciparum variable surface antigen gene families — Supplementary Data 

# DNA secondary structures are associated with recombination in major *Plasmodium falciparum* variable surface antigen gene families

## Supplementary Data

files

**Files in this Data Supplement:**

- Supplementary Data - pdf file
