# Supplementary material for: DNA secondary structures are associated with recombination in major Plasmodium falciparum variable surface antigen gene families
Source: Nucleic Acids Res. 2013 Nov 16;42(4):2270–81. doi: 10.1093/nar/gkt1174 (PMC3936766; doi:10.1093/nar/gkt1174)
Supplement: Supplementary Data [file supp_gkt1174_Supplementary_Material.pdf]

Supplementary material

- **Supplementary table 1 | Primers designed in this study**
- **Supplementary figure 1 | Parent and progeny karyotype and chromosomal positions of *var* gene repertoires of the parental and seven progeny clones**
- **Supplementary figure 2 | Alignments of donor and chimer DNA sequence regions containing recombination sites**
- **Supplementary figure 3 | Density distribution of 50-mer DNA folding free energies in P. *falciparum* real and shuffled *var* genes**
- **Supplementary figure 4 | Examples of *var* 50-mer DNA secondary structures**
- **Supplementary figure 5 | Location of low folding free energy DNA 50-mers in *P. falciparum var* genes**
- **Supplementary figure 6 | Relative location of predicted DNA secondary structures in *var* gene domains CIDR and NTS**
- **Supplementary data file 1 | Fasta file containing chimeric *var* DNA sequences**

Supplementary table 1

Supplementary table 1. Primers designed in this study

a) Quantitative real-time PCR primer pairs for chromosome mapping of *var* genes

Chromosome	Gene name	Forward primer	Reverse primer
Chr.1	PFA0590w	CAGTGGGTTAGTGGTAGTGA	CCTGATGAAGAGGAGGATGC
Chr.2	PFB0490c	TCATGCCCCCTCGACGATGT	CCAAAACTTCCAGTTCGTCC
Chr.3	PFC0615w	GACAACTTCAAGGACATCAAGC	GTTTTAATTCTTTTTGAACACACG
Chr.4	PF3D7_04	CATCAGAACTCCTGCTGCTT	CGGTCATCCTTACCACACAA
Chr.5	PFE0530w	AATACAGCAGGAGCATAATTCC	TCCTTATCATTCTTAGTATTCC
Chr.6	PFF0565c	ACTCCAGAATTTGAAAGATCCTTG	GTTCTTGGAAGCATTGTGCACC
Chr.7	PF07_0073	AAGTAGCAGGTCATCGTGGTT	TTCGGCACATTCTTCCATAA
Chr.8	PF08_0118	GAAACCAAAGCATCGACGAC	GCCATAATTCATGATCGCCG
Chr.9	PFI1500w	ACAAAGACTACACCATTATATCG	TTTCATTTTCTGTATAACTTCCC
Chr.10	PF10_0027	ACTCAGGACGATATTAATCGCT	TGGTGCACTTTCCTTTACCT
Chr.11	PF11_0481	AGCCCGATTGCGGTAGTATGGGT	TCTGTACATAAACGAGCAAAACCA
Chr.12	PFL1510c	TGCCCAATGCTTTGTGCTTGTCC	ACATGATAAAAAATGATCTCTCC
Chr.13	MAL13P1.151	AATTGGGCGTGTGATCCCTT	AGCATCGACTTCGTCTTTCG
Chr.14	PF14_0425	TGTACCACCAGCCTTACCAG	TTCCTTGCCATGTGTTCAAT
	PFA0005w	CTGCTGGTAGTAGTGGTGAGG	CCAGCTAATTCGCTCAAAATTGAT
	PFD1005c	CCATCACGAAGAACAAGACGC	TGGGTGATGTCTTCTGAGGC
	PFD1245c	GGATGACTATAAGGATGCAAAGGAC	GTGCCACCTGTTTCTCCACTAGCA
	PF07_0048	CGGAAACAACTGGCACCGATG	GCGCGCAGGCGCCAACCTCCG
	HB3var2csaB	AGTACAAGCACTATTGGTGACA	TTCCATCAGATGAATTTTGCTG

b) Quantitative real-time PCR primer pairs for *var* exon 1 extremities

var gene		5'-end primer pairs		3'-end primer pairs	
Clone:	Gene name:	Forward primer	Reverse primer	Forward primer:	Reverse primer:
3D7	PF10_0406	GTGCACCAAAAGAAGCTCAA	ACAAAACTCCTCTGCCCATT	GATGATGACGAATCCCTTGAAG	TTGGTTCTCTGTGCCTTCAC
3D7	PFL0020w	TCGATTATGTGCCGCAGTAT	TTCCCGTACAATCGTATCCA	CATGCGGTCCAGATACATCTC	CAGAATGTCACGATTGAACGGTT
3D7	MAL13P1.356	CGGAATTAGTTGCCTTCACA	CATTGGCCACCAAGTGTATC	ACCAACATAGCGACAATCCAC	GTTGGCATATCTCCACAAATCTTC
3D7	PFD1235w	AAACACGTTGAATGGCGATA	GACGCCGAGGAGGTAAATAG	ATCAGAGGGGGAGGCTAAAAC	ATCAAGTTCGCACGGATTTCC
3D7	PFE1640w	AAGAAAGTGCCACAACATGC	GTTCGTACGCCTGTCGTTTA	TTCTGCTTTCAGTCAAGGAC	ATCCACATAACATAGCGTGC
3D7	PF08_0142	GTCGTGGAAAAACGAAAGGT	TATCTATCCAGGGCCCAAAG	AAAGTGGGAAAACCAGCAAT	AAAACACTATCGAAGTTAACGC
3D7	PF08_0107	CCTAAAAAGGACGCAGAAGG	CCAGCAACACTACCACCAGT	CCAGAGGAACCAAAAGAGAC	AGTGAACGCAGCAAAACC
3D7	PFA0015c	TCATTATGGGAAGCACGATT	TGATTTCTACCATCGCAAGG	TGGAACTAATCATCCGAAAAATAC	CAAAATATCCTCCTTGGTATTCC
3D7	PF08_0140	ATGGTGGCAAACTTGTGAGA	TCCAATTGGTCTCCTTGACA	ACACCAAGAAACAACCCAAA	CTTCCCCAACATCTCTGTTC
3D7	PFA0005w	CTGCTGGTAGTAGTGGTGAGG	CCAGCTAATTCGCTCAAAATTGAT	AACTAGTGAAGGCACAGAGAACC	GTTGTGGTTTGGGTTGTGGTTCCA
3D7	MAL7P1.212	TCAGGTTGGTGGGGATGACTAC	GATGCTTGTGACAACAGTCCTGC	GTAGTACCTGAGAAAAAAGTGCC	CTTTTTTTTCTTCACCACCACG
3D7	PFD0625c	CTAGCAAAAGGTTCTGGTGTGG	GGTCTACATCTTCTTTTCCTGTTG	CGATACAACAGAACAACAAGAGG	GTTCGAGGGTTCGTCGTGGTCG
3D7	PF11_0008	GGGTCACAAACATCAAAATTTTC	TTGGCATCTCTCAAATTTCCC	GACGGCTACCACAGAGACAA	CGTCATCATCGTCTTCGTTT
3D7	PFF1595c	CGGGGATGGTATTGAGGATACCACG	CCTGGTGTTTGTGGATCGCTTTC	TATATGTACCGCATGCTCCT	AGGTGTATTTGGTGTATCGC
3D7	PFF0010w	CCGAAGATTCTAACAAGAGTG	CCCCAAAATTGATGAGCTTGC	ACTAATGCGCGTTCAAAATG	CGAACAATGGCAAAACTGTT
3D7	MAL7P1.50	GTGCAGCTCTGATTATACTAAAC	CATGTTTAGCATTACTACTATGATC	GATACACCGTCACCCATTAC	GTGGGATGGGTATTCAATGG
3D7	MAL7P1.55	CCGCCAGGAGGTAGCGTTGGTGC	GTTTGTAGACCCTCTGGTTC	TAGATGAACACGCTTGCAAA	AATAAAAGCGCAATCGAAGTC
3D7	MAL7P1.56	TGCTGATGGTACTGCTAAAAAATA	TTTATTATTCGTACATTCTGCTC	TCTAGTGAAGAACCCGACAA	TAAAAGCGCAATCGAAGTCA
3D7	PF07_0048	CGGAAACAACTGGCACCGATG	GCGCGCAGGCGCCAACCTCCG	TGTGAAGAGAACCACAAACC	TCTTCTGTTTCCTCAAGGGT
3D7	PF07_0050	GAACTGAAGGGAAAATTATGG	CATCTTCTATCACATGGGTC	CCCCAAAACTGCTAATCACA	GACAAAACTCGTCAGTCCAT
3D7	PF07_0051	CACCGATAAACCATGCAATTTTGAT	GCGCCAAAATCCTTATAATGAC	GAAACAGAAGAAAAACCAGAGG	GGGTTTTTCTTTGTCGGTTTCC
3D7	MAL7P1.187	TTTCGGAATTAGCTGGCATC	GCTCCTTCACCATTACTACATTTC	TGACGACGATAAATGGGAAA	TTCTTTTGGAGCAGGGAGTT
3D7	PF08_0103	TCGACGCTAGGAGGTGGTAC	GAAGTAAGCATGGATTTGGTGGC	GTGACATTAACAATAGGTATGAG	GGTGGTTTAACATCATCTGTATC
3D7	PF08_0106	GTAAGGGTGAATTGACAGGACAG	AAACCGGTTTACATCTTCTCC	TTTGTCCGGAAGACGATACA	ATCTGGGGCAGAATTACCAC
3D7	PF11_0007	GCTGCAGATTATAGAAGTTCTTTAC	TAATTGGGAAGCAGTACCAGTATCC	GAGGAAAACACAGTGGAACA	AAGTGAATGCAGCAAAACCA
3D7	PF11_0521	GGGAATGCAATACCAGCGACTCC	CCTATAATCAAGATCACAGGCAC	GATACAGAGGAATCGGCCGT	TTCTCGTGACAATCGTTTTGGTCG
3D7	MAL13P1.1	CTAACATGGAGGCGACAGAAGCAC	GTCAGGTGTAGATGCCTCATCGTCG	ACAAAGGAACGTCCATCTCC	GCCAATACTCCACATGATCG
3D7	PF13_0003	AAAAGAGAGTAAACATGTAAGGC	TAAGCAGACCTTAAATCACCACC	GAAGAAGAAGCACCACCAAAATC	CGCCGTGATCTTCATCTTCCT
3D7	PFA0765c	AAGTATAAAAGTGCCAAAAATGCC	CCTTACTACGATATTGCTCTACAC	ATGACTGTTGTGAACAAGCA	GCAATAGAAGTCAAGGCC
3D7	PFB0010w	TAGTGGGGGCGATAGTTCAC	ATACTCTTGACCAAAACCATG	GATGTATCAACTGCGGGTAG	CCCACACATCCGATATAGGA
3D7	PFC0005w	GGAAAATAGTGCACGAAAAAGC	TATTTCCTGGTGTTTGTTGGC	GAAGATAGTCGATCCGAGGA	AATAAAAGCGCAATCGAAGTC
3D7	PFC1120c	CCGCAAAAGAACCTGACTATAGTAG	TTGTGCTCGCACGGATCAATTACG	GAAAAGGGTGAAGGATCACC	AACCAAGCTGTAAAAGGGTC
3D7	PFD0005w	ATGGTGAGGACAGGAGGTAGCGGTG	TCGGGTTAATATTTGGTGCTTTC	GAAGAAACTGAAAACCCAGTGG	GAATCTGGGGTACTTTCATC
3D7	PFD0020c	GAGGCGATTTATCAAGAGCACG	CAGTAACCCTTATTTTATCACTATT	AAAACAGATGCTCCTCAACC	TTTCTTCAAAAATAAAAGCGCAA
3D7	PFD0995c	GTGGTAGTGGTGGTGTTACTGCTC	GATCACGTATTAATGACTGTGC	CCAGCTGCACCTGATAATAG	TTTCTTTAGTAGAAAGTAACTCAAC
3D7	PFE0005w /MAL13P1.1	GGTATTACTGGTACGCAGGG	TCTATTATATGTACATTGACTTCG	ACTGATAAAGCAGAACGTGG	CAGGGGTGTATTCTTGACAC
3D7	PFI0005w	CAATTTCAGGTCGTTATCGAC	CGCGATTTGCGTCCCACCAAG	CGATAAAGGCCCAGAACAAGC	ACTCCACATGATGGTAGAAGAC
3D7	PFI1830c	AGGATAAGTATAAAAATGCCAAGG	AGGCTGCTAACTGTTTCCCTCC	CACACGTGGACCTCAAGAAC	AAAACCGATGCCAATACTCC
3D7	PFL0030c	GGATAAATCAAGTATTGCTAAC	CATAAGGAGGTGGTATTCTA	GATGATACTTGGACTGATTTGG	TTCAACTTCAGAAATTGCCGACG
3D7	PFL0935c	CAAAGAGTTAAGTGGAAAAATGGGT	ATGGAGCGCAAGCACCACAATTG	GCAGACGAAGACAAATATACG	AGTACCACTTCAATCAACGT
3D7	PFL1960w	GGAACAGGAAAAGAAGACGATTC	ATCATCTAATTTTTTTTTTTTCACC	CAAATAGTGACACACCACCA	GTCCAACGTATGACGTGATG
3D7	PFF1580c	GGTAGTACGCTGGATGAAAG	GTTTGCATGATTCTGACTGC	TAAAAAGCCAGCTGTACCAG	ATCGATCCTAATGCTAACGC
3D7	PF10_0001	AGGTAAAGGAGGTAAGAGTGC	GTCATGATAAGGTATTAATGACTC	TTGTAAACGAGCAGAGGAACCA	ATGCCAGCGCAATACCTAAAG
3D7	PF07_0049	TATTAGTGGTAGTCTTGCTTCTGG	TTCTATATGGAGGACAGGCTC	GAGGGAACAGATGACAAGTG	TTGTTGGTGTTGGTTTGACT
3D7	PF08_0141	AACCTGTGGATACAAGCGATG	CCAAACATATTTCGCTGCCTC	CCTGATTCCCCAATTCTTCC	TTTCTTTATGAAAAGGAACGCT
3D7	MAL8P1.220	GTCCTCTATGTGGAGTGAAAAAGAA	AGTACCGTTATCTGGGTTTATAGGC	CAAAAGCATCCTGTGAAGAATCC	CTTTCTTCTTTTGCAGGTTGTTCG
3D7	PFD1015c	TGCAACGAAACATTAGCACA	AGCAGGGGATGATGCTTTAC	CGCCTGACGAAACTTTTGAC	ATTCGCCTTCTTCATCCACTGC
HB3	HB3var16	CGGTGGTGGAGGTGTTGATAAAG	TGTCGATTCTGTACGACTTGTATC	GAAATAAATTTGATGGTTTGCAGGC	ATCTTTTTGCCCATTGACTTTTTCC
HB3	HB3var48	ACAAAAAGAAGCGCATAAAGATGC	TGACCTGTAGTAACACTGCTTTCA	TACGTTCATGAAAGCACTCACTGC	CCAAAAACACTGACACAAGAAACG
HB3	HB3var2csaB	AGTACAAGCACTATTGGTGACA	TTCCATCAGATGAATTTTGCTG	CCTATGAAACTCTGGAGGAAACTC	GGAACAGGAAAGCGATTGAACTTA
HB3	HB3var08	CGTGTACGACGAAATAGTGGA	CAAGTTGGCATGGACGAGCAC	GATGGTAAAGAAGCTCATCTGTAC	GCTATTGATCCTAATGCCAACG
HB3	HB3var22	CGCAAAAGAACCTGACTATAATAG	CATTATTGTAACCACTGGTAACAG	GCCCTTCTTTCTGCTGCAATG	TATGCCGTATCGTCCACCATAT
HB3	HB3var29	TAGTGGGGGTGCTCATGGTGGC	CAGAAAGTGTTGCAAATGACAC	CGTACACTACAGCTGGAGAC	CTTTCCTCCAGGGCATGTCA
HB3	HB3var13	AAACGTGATGAACTTCTTCGTGC	CATTTTTAGCATTAGTACTATCATC	AGAGTTTCATAAACCAGACGAAACA	TTTCCTGTATCACCCTTGCA
HB3	HB3var1csa	CATTAGAGGGAGAGGCTAGAAGC	GAAATTCTGCTTTCGTTAAATCC	ACATCCTGGTTCATGTTTTGTTGA	CGCTATCAAACCTAACGCCG
HB3	HB3var17	CAGCATCCACTGAAAATAGAAC	GAAACCATCGGTAACATTAGTG	CACCGACTTTATATCACGCCCAC	TTTACCACCATTTTCCACCTTACAC
HB3	HB3var26	CGTGCACCTAGGTATGATC	TCATTTGGTGTTTCTGGATCG	AGATGAGTTCGAAAAGTCAAAGGA	GTTTGACCTGGTTTGCCACT
HB3	HB3var19	CTCGTGGTGGTAGTAGTGGTG	CTGGTGTTGTTTCTTTAGGTC	CAGAACCAGTGGAAGACAAAGAC	GTTTGTTCAGAGTTGGAGCCTT
HB3	HB3var18	ACCTTCACCTGTGCCTAAG	CTGCGATGCCTGAACTCCTTG	AGCTGCAGAATCCCCTAGTAAA	TTGGTTTCGGATTCGGGGT
HB3	HB3var10	GATTTCCACATCGCAATGGGTTCG	TGCTACTATCATTACCCTTTATTCC	TTGGATGTAAATGCGCTAAGAGC	AGGTCAAGGGTTTGTTGTGATG
HB3	HB3var21	GTCGTAGTGCCAGGGATATAC	GTTTTCTGGCGTTGTTCCTATTG	TTTTGATTCCTCCTAGAAGAAGGC	CCAAAATTTGTTTGTGATCTGTACC
HB3	HB3var35	CAACAAGTGCACGAAGAAATAG	ACTACGATATTCCTCTACAAGTG	CACAAACCTAGTGGCGATACC	CTGGGGTACTATCTGCTGGT
HB3	HB3*var2csa* A	GAAAAAATTGAAGAATACTTAAAGG	GTCATTATCGCCATAAGGTAG	AAACAGGCTGGAGGAGACAC	AACTCCCAGACTCACATGCA
HB3	HB3var31	TGGGGATGGTATTGATAAACGAAG	GCAAATCACCATTACTACGTCGTT	ACCTGATGAGGAAGAGGAACTCC	GCCGTTTCTTCACTATTTTCTGC
HB3	HB3var11	GGAATGACTATAGTAAAGCCACTG	GAAACATGCGATGGCACAAATCC	CGAAATGTGCATCTGCTGAATG	TCATGAGCTTCTCTACCAGCTG
HB3	HB3var25	TCGTGGTGGTACGCAGGTTGA	TAGTATGAAGAGAGTGATCAAGAAG	CAACCCACCTGCATATCGAG	TCCATTCTCGAAACCATCGC
HB3	HB3var34	GCAAGGTAGTGGAGGCCAG	TTCTACTCTTGATAAAAACCCATGT	AGACAGCTTAAATCCATGTATGCA	CCGGTTGCATCATCTCCAAA
HB3	HB3var01	GCAGAAATAATAAACAAGGAGGTA	AGCAAGGATTACTAGGAGGTGTCC	AAAGGGGATTGCAATGATGGAAAG	AGCCTCCTGTAAATCACCATCTT
HB3	HB3var27	ATAAAAATGCCCCGGATGCTAAG	GACCCATTTCCTCAACAAAAATTGA	CTTACCTGATGAGGAACCCG	TTCCTTACTAGGTGCTGGTGG
HB3	HB3var28	GTAGCGGTGGTACGCAGGA	GTTCCCTTCAATTTCTCCCTAAAG	TGTGTTACCAAAAACAGAAACAGC	CGCTGGGTCACCATCTTCTTC
HB3	HB3var06	GTATAAAGCAACAGGCAGCTGCTG	GTATTCCATGTATGATTCAAACCAC	TGTCTTCAACAATCAACCAGAAAAG	TGCACAACAAATCCAGTCCAC
HB3	HB3var30	TACGTACAGTAGTGCCAAGGATT	CTGTGAAGGCAGCTGATTCCT	CACCAACAGAACCGGAAGAAAC	TAGGATGGTAGAGTCGAAAGGT
HB3	HB3var12	CAGGTGGGGATGGTATTGATCATGC	TCGCATGGATCTGTAGCTTTGTTG	GGTGGTAGTGAGGGATCATCA	TGGATTCGGTTTCGACCTTG
HB3	HB3var32	TAGTGCTGGTGGAGTGGGTGAA	CCTTTTGCTTCCTGTAACTTTCCT	CCTCCAGCAAAGGTACCTAAAG	CAACGGGAAGGAAGAGATCGA
HB3	HB3var04	GATAGGCACATTATCAAGAGCACA	GATTTTCTTTTCTTCCATGACAAGG	CTTCCTGAAGCTTTCGATTATCCT	TGCTGCAGATTCTACGCAATT
HB3	HB3var36	GCCTACCGCAACAATTGACTATAC	AAACGTTGGTTTTATGACCTCCAG	GATGAACAAAACCAAGTGAAAGCG	TTGATGGAGCTAGGGCACCA

c) Full exon 1 *var PCR* primer pairs and application of amplified fragment

Application	Gene name	Forward primer	Reverse primer	Amplified from genome(s):
Sequencing	PF11_0008	AATGACATTGGAGCGTGTGCCC	CCATTCGGCCCATTCGGTCATC	
Sequencing	PF08_0141	TAATGGTGGTTGCGGAGCCTGT	GGGGGTGCAGGTTTTGGTTCAG	
Sequencing	PFE1640w	ATCATCAGAGGGGGCTAAAAATCC	CCCTGAACCCTAAACCTGAAGATTG	
Sequencing	PF13_0003	TAACATGTAGAGCCCCAAACGGG	GTTGTTCCAAGGATCCAGTGGATG	
Sequencing	PFD1235w	CATCAGAGGGGGAGGCTAAAACC	CTCCTATATTCATTGGACTCTCCG	
Sequencing	PFD0020c	GAGAGGCGATTTATCAAGAGCACG	GGTGGAGGTTCCTCTGTGCCTTC	
Sequencing	PF11_0521	GAATGCAATACCAGCGACTCCGG	TTCTCGTGACAATCGTTTTGGTCG	
Sequencing	PFF0010w	TTTTGGGGGAAACAGCGAGCAC	CACCTGCAGTCGAACAATGGCA	
Sequencing	PFF1580c	TAGCGGTGGTAGTACGCTGGATG	GAAGGAAGGGGTTCTACCTCAGG	
Sequencing	PF08_0140	TTCCCCCTGTCCGTTCCCCTC	GCTAATGGTGGAGGCGCGGGTG	
Sequencing	MAL7P1.187	GGGTGCTGGTGGTAAAAAGGAGG	CTGCTTGGATGGGGAGTAGCTC	
Sequencing	PFD0995c	GTCAGGGTGGGAGTGGTGAG	TCGCTATTATCAGGTGCAGCTGG	
Sequencing	PFD0615c	CAATTTTGGGGGAATCAGCTGGC	AACACTGAAAAGGTCCACACGAGG	
Sequencing	PFD1015c	CGCCGCAAAGTAGTGGGGGTAGTG	TCACCAGGGCGGGTATGACAATGG	
Sequencing	PFL0030c	CTTACGGCCAGAAATGAAGGAG	TTCAACTTCAGAAATTGCCGACG	
Sequencing	HB3 var2csa B	CTTACGGCCAGAAATGAAGGAG	TTCAACTTCAGAAATTGCCGACG	
RFLP analysis	MAL7P1.212	CAGGTTGGTGGGGATGACTACAG	GTGCCGGGGGTGGATCAGAT	
RFLP analysis	PFA0005w	GCTGCTGGTAGTAGTGGTGAGG	GTTGTGGTTTGGGTTGTGGTTCCA	
RFLP analysis	PFD0005w	GCGAATTCATGGTGAGGACAGGAGGTAGCGGTG	GCGCGGCCGCcTGGTGAATCTGGGGTACTTTCATC	
RFLP analysis	PFL0020w	GCTCTAGACGTCTGACTATAAGGATGCCCAGG	GCGCGGCCGCcTGTATCTGGACCGCATGTTCTTCC	
RFLP analysis	PF13P1.356	GCTCTAGAGCAAGGAGGTAGCGGTGATCCG	GCGCGGCCGCcATCTGCTGGTTGTGTTGGTTCAGG	
RFLP analysis	MAL7P1.55	GCCAGGAGGTAGCGTTGGTGC	TCGTCACCTGTTGTTGCCGGTTGC	
RFLP analysis	PFI0005w	GACTCAATTTCAGGTCGTTATCGAC	TCGGCTTGTTCTGGGCCTTTATCG	
RFLP analysis	PF07_0048	CGGAAACAACTGGCACCGATGATC	TGTTTCCGCTTCTGGTGTTGGTGC	
RFLP analysis	PF10_0406	TTGAGGATACAACTGCCAAACATGC	TTTGATCACAGGGGGTTGGTTCTC	
RFLP analysis	PF08_0103	GTCGACGCTAGGAGGTGGTACTG	TTTTGGTCTTGGTGCCACTTTTGG	
RFLP analysis	PFF0020c	GGATCAGATTATTCGTCACCAGGG	GGCTCCAGAACAACTTCTATCAACG	
RFLP analysis	MAL7P1.50	TCGCAAAGTGGTGGAGGTAGTCC	AGTTCCTCTGTGCCTTCACCACTC	
RFLP analysis	PF10_0001	GTAAAGGTAAAGGAGGTAAGAGTGC	TGGTTCCTCTGCTCGTTTACAAGC	
RFLP analysis	PFL0935c	ATGGTGGCAGCGGCCAAAGGTG	TGTCTTCGTCTGCATCACCTTCCA	
RFLP analysis	MAL13P1.1	GCGAATTCACTAACATGGAGGCGACAGAAGCAC	GCGCGGCCGCCACGTTCCTTTGTGCCTTCACTAG	
RFLP analysis	PF08_0106	AGTAAGGGTGAATTGACAGGACAG	CGCACTAGGTGATTCTGGTGGATC	

Supplementary figure 1

a)

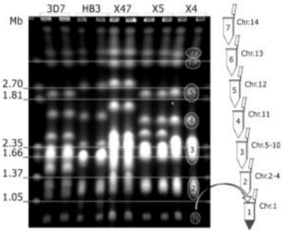

b)

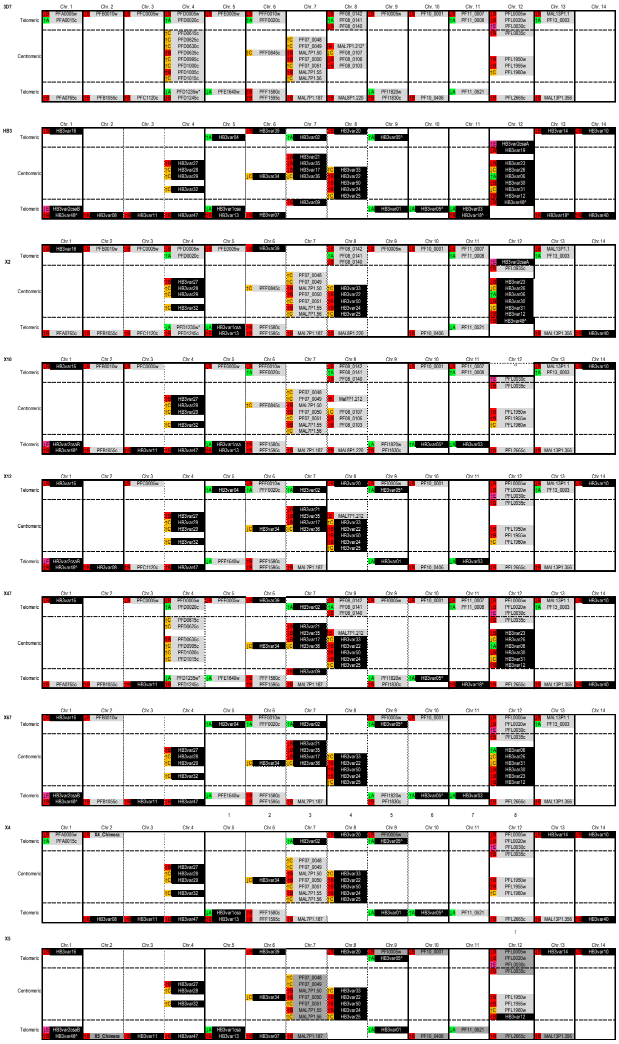

Supplementary Figure 1 | Parent and progeny karyotype and chromosomal positions of *var* gene repertoires of the parental and seven progeny clones

a) Pulsed Field Gel Electrophoresis (PFGE) karyotypes of parent (3D7 & HB3) and three progeny clones (X47, X5 & X4). For illustration chromosome excisions used for X4 var mapping using QPCR are shown. **b)** Chromosomal positions of 3D7 (gray) and HB3 (black) *var* genes and their inheritance in seven progeny clones X4, X5, X2, X10, X12, X47 and X67. The chromosomal orientation (arrows) and *ups* promoter type (*ups*A: green; *ups*B: red; *ups*C: yellow) is shown for each gene. Solid black vertical lines indicate chromosomes within each PFGE separated chromosomal excision. Horizontal dashed lines delimit the centromere and telomere-proximal chromosome regions. Mapping estimates are based on QPCR data and chromosomal locations and orientations of specific genes and *ups*-types from 3D7 and HB3 genome assemblies (PlasmoDB and Broad institute [6,12]. The analysis of the *var* gene segregation in progeny clones illustrates the plasticity of *P. falciparum*´s telomeric regions and that meiosis imposes a Mendelian inheritance of *var* repertoires.

Supplementary figure 2

a

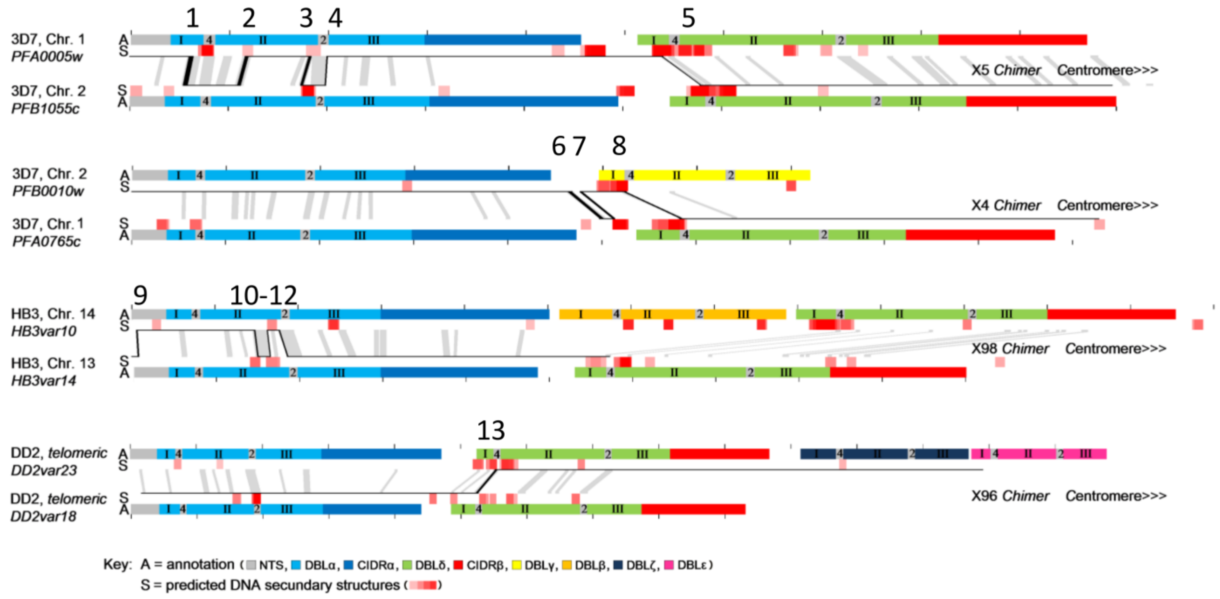

b

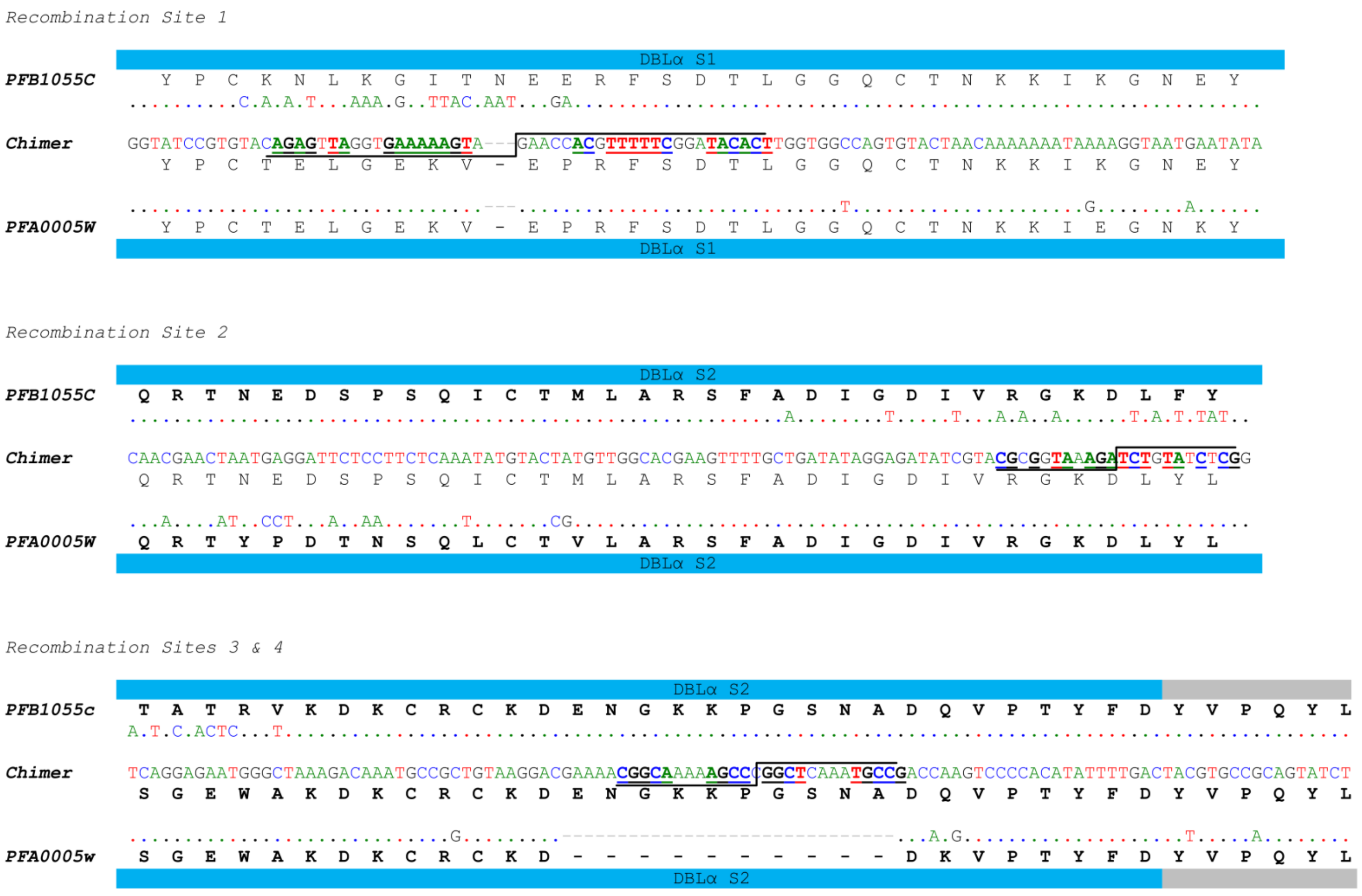

```
            HB2                          DBLα S3
PFB1055c     R  W  F  E  E  W  A  E  D  F  C  R  K  K  K  K  K  L  E  K  L  E  Q  Q  C  R
            ...................................GAA..AA...A.G......G..A.GTTGG..C.AC.G.....C

Chimer      TCGCTGGTTCGAGGAATGGGCAGAAGACTTTTGTAGATTAAGGAAACATAAATTAAAAGATGCTAAAAACAAATGTCGT
             R  W  F  E  E  W  A  E  D  F  C  R  L  R  K  H  K  L  K  D  A  K  N  K  C  R

            .....................C.....T..................................................
PFA0005w     R  W  F  E  E  W  A  E  D  F  C  R  L  R  K  H  K  L  K  D  A  K  N  K  C  R
            HB2                          DBLα S3
```

Recombination Sites 5

```
            DBLδ S1                                                                  HB4
PFB1055c     W  K  C  V  S  S  G  E  K  S  V  A  T  A  G  S  S  G  A  T  G  K  S  G  D  K  G  A  I  C  V  P  P
            .....A.....AT..AGTGGTGAAAAAGTGTTG.C.C..CCGGT.GT....GTG.............................................

Chimer      TGGAAGTGTGTCACA------------------CCAAGTGGTAACACGAGTGACACCACTGGCAAAAGTGGTGATAAGGGTGCCATTTGTGTGCCACCC
             W  K  C  V  T  -  -  -  -  -  -  P  S  G  N  T  S  D  T  T  G  K  S  G  D  K  G  A  I  C  V  P  P

            ...............------------------........................T...........C.CCACC.GTGG.A..A.TTGTGTG
PFA0005w     W  K  C  V  T  -  -  -  -  -  -  P  S  G  N  T  S  D  T  T  V  K  S  G  D  T  T  G  G  S  I  C  V
            DBLδ S1                                                                        HB4
```

RECOMBINATION SITES 6 & 7

```
                                               inter domain region
PFB0010W     I  T  H  H  D  G  E  H  S  S  D  E  D  E  E  E  E  E  E  E  E  Q  Q  -  -  P  P  A  E  G  T  E  Q  G  E  E  K
            .............................................................A......C.GC..------C.GCCG..G.AA...ACG..ACAGGG........

Chimer      ATCACCCACCACGACGGCGAACACTCCTCAGACGAAGACGAAGAAGAAGAAGAAGAGGAGGAGGAAAAGGAAAACGCCGACGCAGGCGGCGAAGATGGCAAGGAGGAGAA
             I  T  H  H  D  G  E  H  S  S  D  E  D  E  E  E  E  E  E  E  E  E  K  E  N  A  D  A  G  G  E  D  G  K  E  E  K

            .A...TG.GG.T....A..CCG.AGAAGAC..A.............................................................................
PFA0765C     N  T  E  D  D  D  A  E  E  D  E  E  D  E  E  E  E  E  E  E  E  E  K  E  N  A  D  A  G  G  E  D  G  K  E  E  K
                                               inter domain region
```

```
PFB0010W     S  E  S  K  E
            ...............

Chimer      GTCGGAGTCCAAGGAG
             S  E  S  K  E

            ......GAA......
PFA0765C     S  E  E  K  E
```

RECOMBINATION SITE 8

```
            DBLγ S1                                 HB4                                                  S2
PFB0010W     I  A  P  S  G  T  T  S  G  K  D  G  A  I  C  V  P  P  R  T  Q  E  L  C  L  Y  Y  L  K  E  L  S  D
            ...................................................AC.A.C..A.G..T...G.T..T.TT.TT..AA...AT.GAGTGAC

Chimer      TATCGCACCTAGTGGTACCACTAGTGGAAAAGACGGTGCTATATGTGTGCCACCCAGGAGACGAAAACTATACATACAGAAACTACCAGACGTCGAATTT
             I  A  P  S  G  T  T  S  G  K  D  G  A  I  C  V  P  P  R  R  R  K  L  Y  I  Q  K  L  P  D  V  E  F

            C.C.C...........T---------..CC.G.....C..............................................................
PFA0765C     T  P  P  S  G  T  -  -  -  N  Q  G  A  I  C  V  P  P  R  R  R  K  L  Y  I  Q  K  L  P  D  V  E  F
            DBLδ S1                                 HB4                                                  S2
```

RECOMBINATION SITE 9

```
                                                  NTS
HB3var14     M  V  T  L  G  G  G  G  G  S  S  G  D  A  E  K  Y  K  Y  V  N  D  A  K  H  L  L  D  M  I  G  E  T
            ...................................A..C...GA.GTA.A.GT.TGT.AACGA...T..G...C.T.......TG..T..AG...CT.

            ATGGTGACGCTAGGAGGTGGTGGTGGAAGTAGTGGTGGTGAGGATGATATTGATAAAACA---AGTGCCAAACATTTATTGGATAGCATAGGGAAAATAG
Chimer       M  V  T  L  G  G  G  G  G  S  S  G  G  E  D  D  I  D  K  T  -  S  A  K  H  L  L  D  S  I  G  K  I

            .........A.A.T.........CTG.....................................---...................................
HB3var10     M  V  T  Q  S  G  G  G  A  G  S  G  G  E  D  D  I  D  K  T  -  S  A  K  H  L  L  D  S  I  G  K  I
                                                  NTS
```

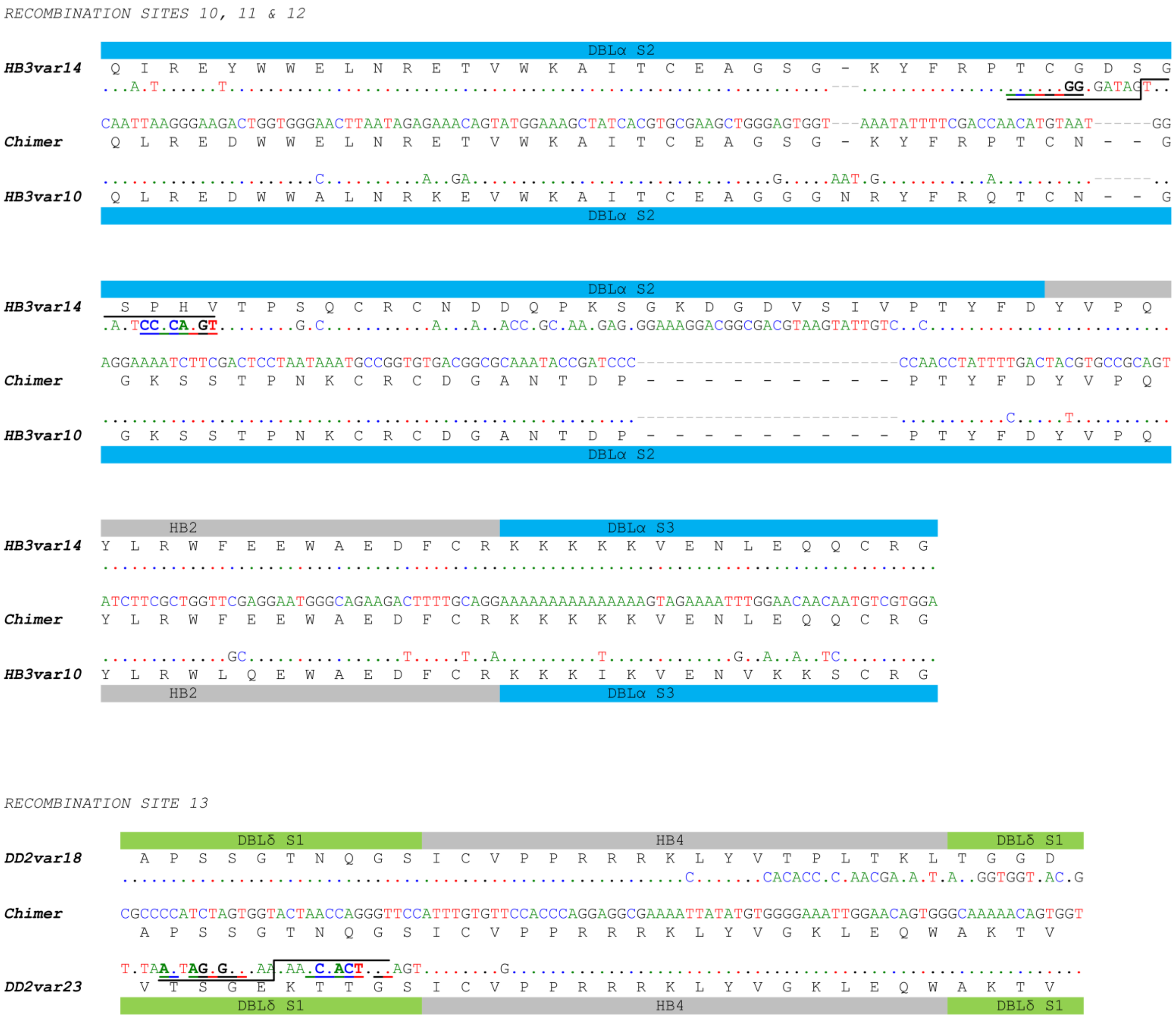

Supplementary Figure 2 | Alignments of donor and chimer sequence regions containing recombination sites

a) Schematic presentation of Figure 1 showing numbered recombination sites. **b)** Alignments of DNA sequence regions surrounding each of the 13 observed recombination sites with annotated domains of the encoded protein. Dots indicate base pairs identical to the chimeric sequence. Examples of quasi-palindromic sequences are highlighted by bold and under/over-lined formatted nucleotides.

Supplementary figure 3

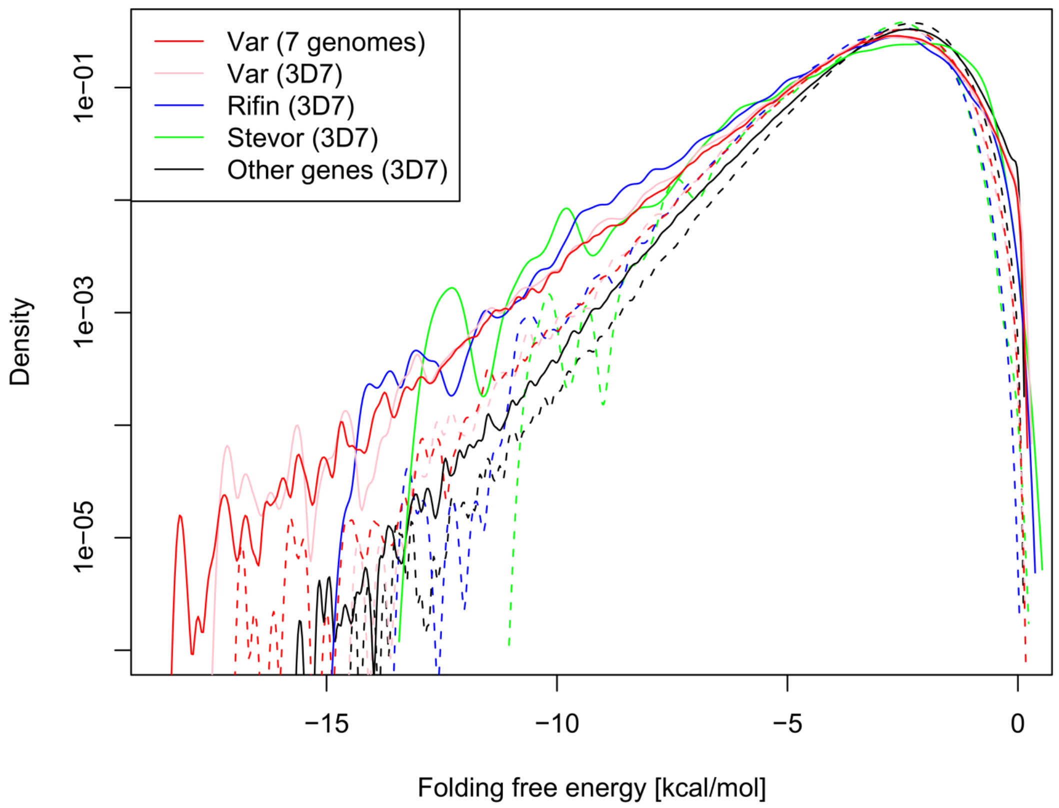

Supplementary figure 3 | Density distribution of 50-mer DNA folding energies in *P. falciparum* real and shuffled genes

The distribution of 50-mer folding free energies in each of five *P. falciparum* gene groups is plotted with solid lines, while dashed lines show the distribution of 50-mer folding free energies in the same gene groups with each gene sequence randomly shuffled. The folding free energy reflects the probability of a DNA 50-mer to form a DNA secondary structure (DSS); with lower folding free energy 50-mers having higher likelihood of forming DSS. The *var* genes contain the 50-mers with the lowest folding free energies, but all telomeric antigen gene families (*var, rif* and *stevor*) contain significantly higher proportions of 50-mers predicted to form DSS (folding free energy cut-off set at <-6.27 kcal/mol) compared to the group of all other *P. falciparum* genes (p<2e-16 for all comparisons). In *var*, *rif* and *stevor* genes, the proportion of 50-mers with folding free energy <6.27 kcal/mol is 5.6%, 8.2% and 5.6%, respectively. For comparison the proportion of the group of all other *P. falciparum* genes is 2.1%. In all *P. falciparum* gene groups the folding free energy of 50-mers is lower than would be expected from the nucleotide composition alone.

Supplementary figure 4

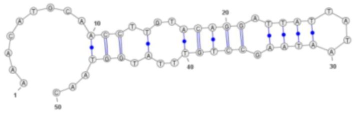

DSS near recombination site 1:
PFB1055w 176-225

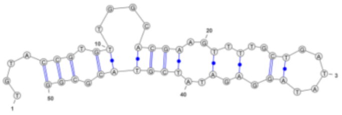

DSS near recombination site 2:
PFA0005w 571-620

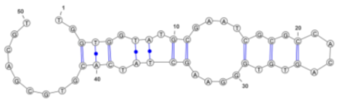

DSS near recombination site 3:
PFB1055w 757-806

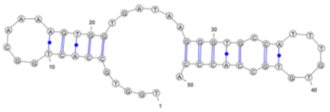

DSS near recombination site 5:
PFB1055w 2886-2935

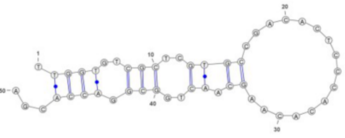

DSS near recombination site 6:
PFA0765c 2390-2439

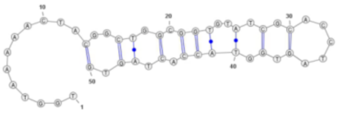

DSS near recombination site 7:
PDB0010w 2550-2599

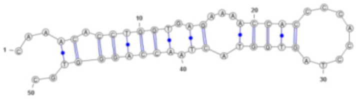

DSS near recombination site 8:
PFA0765c 2863-2912

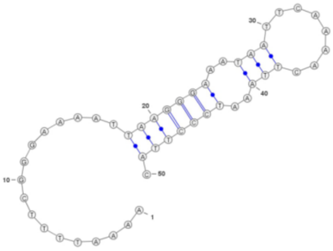

DSS near recombination site 10:
HB3var14 676-725

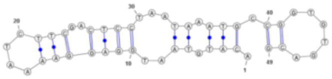

DSS near recombination site 11:
HB3var10 817-866

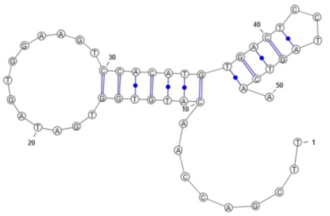

DSS near recombination site 12:
HB3var14 815-864

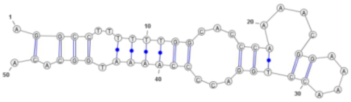

DSS near recombination site 13:
DD2var23 3392-3441

Supplementary figure 4 | Examples of *var* 50-mer DNA secondary structures
Examples of minimum free energy structures of *var* 50-mers predicted by RNAfold. 50-mers, which are located near recombination sites, and that have a high propensity of forming secondary structures are shown.

Supplementary figure 5

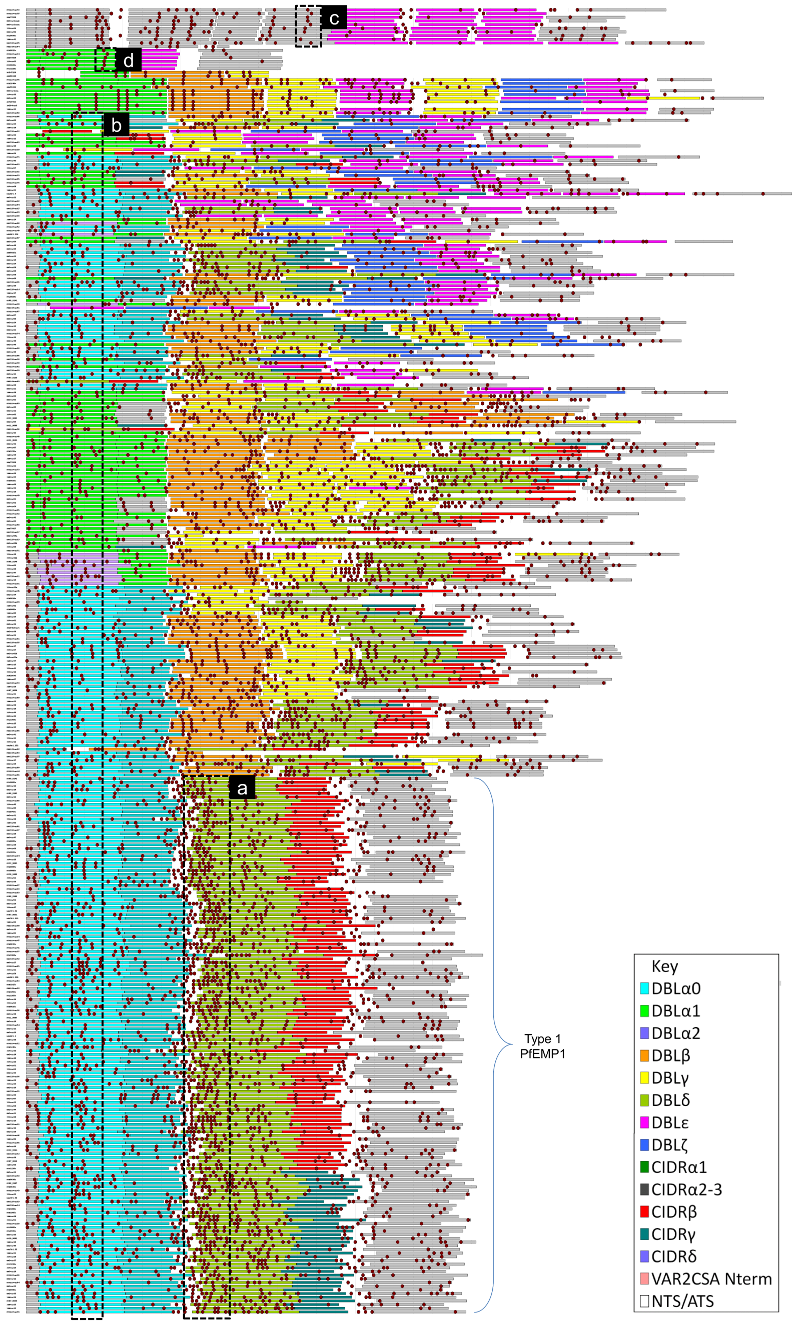

Supplementary Figure 5 | Location of low folding free energy DNA 50-mers in *P. falciparum var* genes

The DNA 50-mers in var genes with the highest potential to form DNA secondary structures (DSS) (folding free energy <-6.27 kcal/mol) are shown as brown dots superimposed onto of 366 domain annotated PfEMP1 sequences from seven different P. falciparum genomes (31) originating from Central America (HB3), Africa (PFCLIN, IT/FCR3) and Asia (RAJ116, IGH, DD2). 3D7 is the Schiphol airport canal isolate. For simplicity, only non-overlapping 50-mers are plotted. PfEMP1 are aligned by composition of phylogentically distinct DBL and CIDR domain sub-classes flanked by N-terminal (NTS) and C-terminal acidic segments (ATS) (see colour code). Frames indicate previously identified recombination hotspots, which are particularly enriched for DSS. These include hotspots in ´mid var gene inter-domain region´ (a) [6,7], the boundary between DBL subdomain 2 and 3 (b) (31), DBL3 of var2csa (c) (47) and (d) the first DBL of var3 (31). Plots using varying folding free energy thresholds yield similar results as those presented here.

Supplementary data file 1

Fasta file with chimeric *var* sequences

>X4PFA0765c_PFB0010w
TAGTGGGGGCGATAGTTCACGGGATGAAAGTGTCAAAGATTTATTTGATAGAATAGGGAAGAA
AGTTTACGAAAAAACAGAAAAGATTGCAAAACGATATACTACTGAATTGCATGGTGATTTGTC
AAAAGCAACATATCCAAATGATAAACATCCTGAAGGATCAACAGAAAATAATCCATGCAAACT
TCAATATGATTATAATACTAATGTTACTCATGGTTTTGGTCAAGAGTATCCTTGTGAAACGGAC
ATAGTAGAACGTTTTTCTGATACAGAAGGAGCACAATGTGATAAGAAAAAAATAAAAGATAAT
AGTGAAGGAGCTTGCGCTCCATATAGACGATTACATGTATGCGTTAGAAATTTGGAAAATATCA
ATGATTATAGTAAAATTAATAATAAACATAATTTATTGGTAGAAGTGTGTCTTGCAGCCAAATA
TGAAGGGGAATCAATAACAGGTCGTTATCCACAACATCAAGAAACTAATCCTGATACTAAATC
TCAACTATGTACTGTATTAGCACGAAGTTTTGCAGATATAGGTGATATTATAAGAGGAAAAGAT
CTGTATCGTGGTGGTAATACCAAAGAAAAAAAAAAAAGAAAAAAATTAGAAGAAAATTTAAA
AACGATTTTCGGGCATATATATGATGAATTGAAGAATGGGAAGACGAATGGGGAGGAGGAGCT
ACAAAAACGCTACCGAGGTGATAAAGATAATGATTTTTATCAATTACGAGAAGATTGGTGGGA
CGCTAATCGAGAAACGGTATGGAAAGCTATCACATGCAACGCTGGAAGTTATCAATATTCTCA
ACCAACATGTGGTCGTGGAGAAATTCCATATGTGACGCTTAGTAAATGCCAATGTATTGCTGGA
GAAGTTCCTACATATTTTGACTATGTCCCACAATATTTGAGATGGTTCGAGGAATGGGCAGAAG
ACTTTTGTCGTAAAAAAAAAAAAAAAAATACCAAACGTTAAAACAAATTGTCGTCAGGTACAGA
GGGGTAAAGAAAAATATTGTGATCGTGATGGATATAATTGTGATGGTACTATTAGAAAGCAAT
ATATTTATCGTTTGGATACTGATTGTACTAAATGTTCTCTTGCATGTAAGACTTTTGCGGAATGG
ATTGATAACCAAAAAGAACAATTTGACAAACAAAAACAAAAATATCAAAATGAAATATCAGGT
GGTGGTGGTAGGAGGCAAAAACGGAGTACACATAGTACTAAAGAGTATGAAGGATATGAAAA
ACATTTTAATGAAGAACTACGAAATGAAGGCAAAGATGTGAGAAGCTTTTTACAATTGTTAAGT
AAAGAAAAAATATGTAAAGAAAGAATTCAAGTAGGAGAAGAAACAGCAAATTATGGTAATTT
CGAAAATGAAAGTAATACTTTTTCTCATACGGAATACTGCGATCGTTGTCCTTTGTGTGGAGTT
GATTGCAGCAGTGATAACTGTAGAAAAAAGCCAGATAAATCATGCGATGAACAAATTACTGAC
AAAGAATACCCTCCTGAAAATACTACGAAAATCCCAAAACTTACCGCTGAAAAAAGAAAGACA
GGTATACTAAAGAAATATGAAAAGTTTTGTAAAAATAGTGATGGTAATAACGGTGGTCAAATT
AAAAAATGGGAATGTCATTATGAAAAAAATGATAAAGATGATGGCAATGGTGACATTAATAAT
TGCATACAAGGAGATTGGAAAACTTCTAAAAACGTATATTACCCTATATCCTATTATTCATTTTT
TTATGGTTCGATTATTGATATGTTAAACGAATCTATTGAGTGGAGAGAAAGACTTAAGAGTTGT
ATAAATGATGCGAAATTAGGAAAATGTAGAAAAGGATGTAAAAATCCATGTGAATGTTATAAA
CGATGGGTTGAAAAAAAAAAAGACGAATGGGACAAAATAAAAGAATTTTTTCGCAAGCAAAA
AGATTTGCTAAAAGACATTGCAGGAATGGATGCTGGCGAACTTCTTGAATTTTATTTGGAAAAT
ATTTTTTTGGAAGATATGAAAAATGCTAATGGAGATCCAAAAGTAATAGAAAAATTTAAAGAA
ATTTTGGGAAAGGAAAATGAGGAAGTTCAAGATCCTTTAAAAACGAAAAAAACAATTGATGAC
TTTCTCGAAAAGGAATTAAACGAAGCCAAAAACTGCGTAGAAAAAAATCCAGATAACGAATGT
CCAAAACAAAAAGCCCCCGGCGATGGTGCCGCCCCCTCCGACCCACCACGTGAAGACATCACC
CACCACGACGGCGAACACTCCTCAGACGAAGACGAAGAAGAAGAAGAGGAGGAGGAAA
AGGAAAACGCCGACGCAGGCGGCGAAGATGGCAAGGAGGAGAAGTCGGAGTCCAAGGAGGTG
GTAGAACAACAAGAGACACCACAAAAAGACACAGAAAAGACGGTACCAACAACAACACCAAC
AGTAGACGTTTGCGACACAGTGAAAACCGCACTCGCGGACACGGGCAGTCTCAATGCTGCATG
TTCCCTCAAATATGTTACTGGTAAAAACTACGGCTGGCGGTGTATCGCACCTAGTGGTACCACT
AGTGGAAAAGACGGTGCTATATGTGTGCCACCCAGGAGACGAAAACTATACATACAGAAACTA
CCAGACGTCGAATTTGACGACAAGTCATTACGTAAGTGGTTTATAGAGACGGCAGCTATAGAG
ACTTTTTTCTTATGGGATAGATATAAAAAAATAAAAGCAAAAGAGAAAAAAGAAAAAGAGGAT
GCAAAAGGACAAATATATGAATCAGCCGGTGAAGACGACGAAGACAAAGACCCCCAAGAGGA
ATTACAACGTGGAGATATACCTGATGGTTTCTTGCGGCAGATGTTTTATACATTAGGAGATTAT

AGAGATATATTAGAAGGGAAAAACGATATCCTAATTGGAAAAACAGGTACTGGTAGTGCAAAA
GATGAAATGGCTGATAAAGAAAATAAAATAAAAGAAGCTATAAAAAAGTTTTTTCAAAATGGT
GATTCCCAACTTCCTAGTGGAAAACCTGGTGATGAACGCAAAAAATTTTGGGAAGCAAATGAA
AAACATATATGGAATGCTATGGTATGTGCTCTAACCTATGAAGAGAAGACAAGTAGTGCTAGT
GGTGGAGAAAAAAACACAACTATTACACAGGACGATGGTTTGAAAGGTGCACTTATCAAAGAT
GGCAATCCCAAAAACCCCCAATACCATTACGAAAAAGTGACACTCGAAAATAGTGGTCCCAGC
CCCAAACTCCAAACCGGCTCCCCCGGCACTAGTGGTGATAACACCCCCCTCACCCAATTTGTGA
AAATACCTACATTTTTTCGTTGGTTACATGAGTGGGGAAGCGACTTTTGTGGTACGCGGAAGAG
GATGTTGGATAAAATAATATTTGAATGTCGTGGTAATGGAAAGGTTTGTAGTGGAGATGGGGA
GGACTGTAAAGATCAGCTTAAACACAATCCTAGTACTGTTCGGGATTTTTTGTGTCCGACGTGT
GGCAGACATTGTAGTTTTTATAAAAAATGGATAGACATAAAAAAAAAGGAATTTGAGGAACAA
TCAAATGCATATACTGGACAAAAAGACAAATGCAAAAAGGATAGTAATAATGGATTTTATAGA
AAACTACAAACATATAATGAAGCTAAAGACTTCTTACAAACGTTAAGACCATGTAAAACTAAT
AATGAGAATAATAATGGAGATGATAAATTAGATTTTACAAATCCAAAGGAAACATTTAGACCT
GCGAAAAATTGTAAACCATGTTCTGAATTTAAAGTTAAATGTGATTATGATAATTGTACTGGTG
CTAATGCAAATACGTGCACTACACGAAAGATTTCTCCAAAAGATATTGACGAAAAGACAGATC
CTAATGGAAACATAGAAATGCTTGTGAGTGATGACAGTGCAACGGGATTTGCAGGTGATTTAA
ACGATGATTGTAAAAATGCACATATTTTTAAAGCTTTTAGAGAAGATGTATGGACATGTGGTAA
GGTATGTGGTTATAATGTATGTAAACCGGTAAAAGTTAATGGGGAAACATTCGATGCAAAAAA
AAAAGGCGAAAACCAAATTATAATTATTAGGGCATTGTTTAAAATATGGTTAGAATATTTTTTA
GAAGATTATAATAAAATTAAAAAAAAATTAAATCCATGTAGGAATAATGGTGAGGTATCCAAA
TGTATAAATGATTATGACGAAAAACATAAATGTGTAGAACAATGGATAGAGAAGAAAAGAGC
AGAATGGGAAAAAATAAAAAAACATTACAAAAGACAAAATGAAAAGGGTGACACAGAAATGA
TATCTTTGGTTAGAAATTTTTTGGGTGACGTGCAACCCCAAACTGAAGTTCACAAAGCTATACA
ACCTTGTAAAGATTTAGATAAGTTTCAAGATTCAAGTTATTGTACTGTCAATGGGAGCTCAGCA
AAAGGTAAAGACGGTACACAAAAAGATATCGTAGAATGTTTGTTTCAAAAGCTTGAAGAAAAA
GCAAAAACGTGTTCCACCTCAACTAGTGAAGAAACCCAAAACACAGCACAGTGTCAAGATACC
CACCCTGATGATGACTTATCCCTTGAAGAAACAGAGGAGGTGAAGGCGCCAAACATTTGTCCA
AAAGACCCTGAATCAAAAAAAGATGAACCAGATGACTGTTGTGAACAAGCAAAGGCACTACCA
AAAGAAACAGCGGATGTAGTAGCAGATAATGGTAGTGGAAACGATGAAGTACAAGAGGAAGA
AGA
ATCCGAAGAAAAAAATAAAGGAGATGCTGCCCCATTACCGGAAAAACCATCAGGTGATTCCAC
CTCTACCGACCAAACCGAACCCCCAGCACCAGCACCACAACCACTCCCAAGTGATAATACCAG
CGATATATTAAAAACTACTATCCCGTTTGGTATTGCTTTGGCCTTGACTTCTATTGC

>X5PFA0005w_PFB1055c
TGCTGCTGGTAGTAGTGGTGAGGAAGATGCCAAACATGTATTGGATGAATTTGGGCAACAAGT
GTACAATGAAAAAGTGGAAAAGTATGCTAATTCTAAAATATATAAAGAGGCGTTGAAAGGAGA
TTTGTCACAAGCATCAATTTTGAGCGAATTAGCTGGCACCTATAAACCATGTGCCCTTGAATAT
GAATATTATAAGCATACTAATGGCGGTGGTAAGGGTAAAAGGTATCCGTGTACAGAGTTAGGT
GAAAAAGTAGAACCACGTTTTTCGGATACACTTGGTGGCCAGTGTACTAACAAAAAAATAAAA
GGTAATGAATATAGTACTAAAAGTGGTAAAGATTGTGGAGCATGTGCACCATACCGACGTCTA
CATTTATGTAGTCATAATTTGGAATCTATAGACACAACGTCGATGACGCATAAGTTGTTGTTAG
AGGTGTGTATGGCAGCAAAATACGAAGGAAACTCAATAGATACACATTATCCACAACATCAAC
GAACTAATGAGGATTCTCCTTCTCAAATATGTACTATGTTGGCACGAAGTTTTGCTGATATAGG
AGATATCGTACGCGGTAAAGATCTGTATCTCGGTAATCCACAAGAAAGTACACAAAGAATAAT
ATTAGAAAATAATTTGAAAGATATTTTCGCGAAAATACATAGTGACGTGATGTCAACGAGCGG
GAGTAATGGGAGGGCGCTACAAAAACGCTACAAAGATACTGATAATTATTATGAATTGAGAGA
AGATTGGTGGGCACTTAATAGAGACCAAGTATGGAAAGCTATCACATGCAATGCTGGGGGTGG
TAATAGATATTTTCGACAAACATGTGGTTCAGGAGAATGGGCTAAAGACAAATGCCGCTGTAA
GGACGAAAACGGCAAAAAGCCCGGCTCAAATGCCGACCAAGTCCCCACATATTTTGACTACGT
GCCGCAGTATCTTCGCTGGTTCGAGGAATGGGCAGAAGACTTTTGTAGATTAAGGAAACATAA
ATTAAAAGATGCTAAAAACAAATGTCGTGGAGATAGTGGTAACGATAGATATTGTGATCTTAA

TAGGTATGATTGCACACAAACTATTAGAGGAAATGAACATTTTGTTGAAAAGGATGATTGTAA
AGGTTGTCAGTATTCGTGCGCTCATTTTGTGAACTGGATAGATAACCAAAAACTAGAATTTGAA
AAACAAAAAGAAAAATATACAAAAGAAATTAAAAAAAAGCATCCAACAACCATAATAATAAA
AACTGCAAATCGAAAAACAACTATTAATAACTTATATGTAAAAGAATTTTATAAAAAACTTCAA
GAGAAATATGGAGATGTCGAAAATTTTTTACAAAAATTAAATGAAGAACAAATATGCAAAAAT
CAACCGTACAATGATGAAAGTAGTATTGATATTAATTTCAAAAGTATTAAAGATATTGACATAT
TTTCTCATACGGAATACTGTCAAGCATGTCCATGGTGCGGGGCCAAACGTAAAGGTAAAGGAT
GGGAACCTAAAGAAAAAACCTGTGGAAAAACAAAGACATACGATCCTAAGAAAACAACGAAT
ATACCAATACTTACCCCTTATATATCACAGCAAAGTATACTAAAAAAATATAATAAATTTTGTA
ATGGTAATGGTGGAAATGGTGCACCTGCTACTGCAACTGGTGGTGGTCAAATTAAAAATTGGC
AATGTCATTATGAAGGTGATAATAATGATAATTGTGTAGAAGGAGAATGGAAAGAGTTTAAAG
AGGGTAAAAACGTTATGTCCTATAATGCTTTTTTTTGGAAGTGGGTTCATGATATGTTAATCGAC
TCTATGCAATGGAGAAATGAACATGGAAATTGTATAAATAAAGATAATGACAACACATGTAAA
AATTCATGCAAAAGACCATGTGAATGTTTTAAAAGATGGGTAGATCAAAAAAAAAAAAAACGAA
TGGGAGGCAATAAAAGACCATTTTAAAAAGCAAAATATTGCAGCTGAAACACAATGTGATCCT
GGCGTAACTCTTCAATGGGTTTTGATATTAGACTTTTTGAAAGACGAATCCACAGAAGATAAGG
AAAATAAGGTGAGTGCAGAGGAGGCAAAGGAAATAAAACACCTTCGCCAAATGTTGCAACAA
GCAGGCGTTGATGATCCTGCTGCTTTTGCTCGTCCGTGTACTGAAGATGGTGTCGCTGAACAGG
ACACTATAATGGATAAATTGCTCAATCGCGAAGAAAACGATGCCACTGAATGCAAAAAATGCG
ACAAACCACCACCAGCACCCACTGCAGGAGATCGTGGCCCTGGAGCCCGCGCCGACCCCCACG
ACGTCCAACAGCCACGACCTCCTGGTAGTGGCCCCGGCACGGACGCCAACGACGAAGACGATG
ATGACGATGATGACGATGATGACGAAGAAGACGGTGAAGCCAAAGAAGAAGAAGAAGACGAG
GAAAAACAAGAGGACGTCCACCAGGAGGAAAAGGCAAAGAAGGAAGAACCACAAAAAGAGG
AGGTGGCACGAACACCAAAAGACGATGTAAATGTGTGCAATATAGTGAACAATGTGTTTACAG
ACGGCAGTAGTCTCCAAGCAGCGTGCTCTCTCAAATATGGCAAAAACGCACCCACAAGTTGGA
AGTGTGTCACACCAAGTGGTAACACGAGTGACACCACTGGCAAAAGTGGTGATAAGGGTGCCA
TTTGTGTGCCACCCAGGAGGCGACGACTATACGTGGGTGGGTTAACCAAGTTGACAAGTGCTG
GCACGTCTAGTGAGTCACCACAGGGGGGTAGTGAGTCATCACGGGCGAGTGATGTGTCACAAG
GTAACGGCGGCGACGACATCACCACCACCGAGTCATTACGTAAGTGGTTTATAGAGACGGCAG
CTATAGAGACTTTTTTCTTATGGCATAGATATAAAAAAGAGTGGGAGGCACAAAAGAAGGCGG
AACTACAACGAAATGGATTACTACTCGGCACAGGTGCTAGCCTCAACCTTGGTGGTGATGACTC
CAACCCCCAAACACAATTACAAAAAAGTGGTACCATACCCCTCGATTTCTTGAGATTAATGTTT
TATACTTTAGGTGATTATAGAGATATTTTGGTACGAGGTGTTGCTGACGACAAAAACGGTGGCA
ACAACATAATACTTAATGCGAGTGGTAACAAGGATGAAAAACAGAAAATGGAGAAAATACAA
GAGAAAATAGAACAAATTCTTCCAACTAGTGGTAACAAAGAAACTCGTGGCCCCCAAAATAGT
GTCAATGACCGTCAATCCTTGTGGGATAGAATCGCCGAACATGTTTGGCATGGAATGGTTTGCG
CATTAACATATAAAGATGACGACAATGGCCTCAAAGGCGTCGTAAAAAAACCACAAAAGATTG
AAAATCCGGAGAAACTTTGGAACGAAACAACCAAAAAACCCAAAGACGAGAAATACCAATAC
CAAACTGCCAAACTCGAAGATGAAAGTGGCGAAAAACGACCAGACTCCTCAGCCAGTGGTACG
AAATTAACCGACTTCATCAAACGCCCCCCTTATTTCCGTTACCTTGAAGAATGGGGTCAAAATT
TTTGTAAAAAACGAACAGAGATGTTGGGGAAGATAAAGGAGGAT
TGCTACAAAAATGGTGGACGTTGTAGTGGTGATGGTTTGAAATGTAACGAAATAGTTATAGATA
AGGAAAAAATTTTTGGCGATTTACTTTGTCCGACGTGTGCCAGACATTGTAGATTTTATAAAAA
GTGGATAAACACAAAAAGGGACGAATTTAATAAACAATCAAATGCATATTCTGAACAAAAAAA
AAAATACGAAGAGGAAAATGATAGTGCTCAAAAGAATAATGGAGTTTGCGGAACACTAAAAG
ATGACGCTGCAGAATTTTTAAATAGGTTAAAAAACGGACCATGTAAAAATGAGAGTGAAGAGA
ATAAAAAAGCAGAGGATGAAATAGATTTTAAGAAACCAGATGATACATTTAAAGATGCAGATA
ATTGTAAACCATGTTCTGAATTTAAAATTAAATGTGAAAATCATAATTGCAGCAGTGGTGGTAA
TACACAAGGGAAGTGCGATGGAAAAACGACTATTGCTGCAACAGAAATTGAAAATATAAAAAC
AAATACTAAAGAAGTTACTATGCTTGTGAGTGATGACAGTAAAAGTGCAACGGAATTTAAGGA
TGGTTTAAGCGAATGTAAAGATAAAGGTATATTTAAAGGTATTAGAAAAGATGAATGGGAATG
TGGCAAAGTATGTGGTGTAGATATATGTAATCTGAAAAAAAAAAGATAACATTGGGAAAGAAGC
GATAAAAAATATATCATAATGAAAGAATTGCTTAAACGATGGTTAGAATATTTTTTAGAAGATT

ATAATAAAATTAAACATAAAATTTCACATTGTACGAAAAATGGTAAAGGATCCAAATGTATAA
AAGGTTGCGTAGATAAATGGGTACAACAGAAAAAGGAAGAATGGAAACAAATAAAAGAACGT
TTCAATGAACAATATAAAAGTAAAACCTCAGATGAATATTTTAACGTTAAAAGTTTTTTGGAGA
CCTGGATACCTAAAATTGCTGTTGTAAATGATCAAGATAATGTTATAAAATTAAGTAAGTTCGG
TAATTCTTGTGGATGTAGTGCCAGTGCGATCTCAACAAATGGTAATGAGGAGGATGCTATAGAT
TGTATGATTAAAAAGCTTGAAAAAAAAAATTGACGAATGCAAAAGGAAACCTGGCGAAAATAGT
GGTCAAACATGTAACGAAACACTAACACATCCCCTTGACGTTCAGGATGAAGATGAACCCCTT
GAAGAAACAGAAGAAAACCCAGTGGGAAAACAACACCCATCATTTTGTCCGCCAGTGGAAGAT
AAAAAAAAAGAGGAAGAAGGAGAAACTTGTACACCGGCATCACCAGCACCAGCACCAGCACC
AGCACCAGCATCTCCATCCCCGACACCGGCCCCTGCGGATGAACCGTTTGACCCAACTATACTA
CAAACAACCATTCCTTTAGGTATTGCGCTGGCATTAGGATCCATTGCTTTTTTATTTTTGAAGGT
AATATATATATGTGTGGTATATATGTATATATATATGTGTTTCTGTATATATATGTATGTGTGGG
TGTGTTTGGATATATATATATGTGTATGTATAAGTGTTTGTGTATATGTATGTGATTTATATATA
TTTTATATA

>X96DD2var18_DD2var23
TGCTGGTAGTAGTGGTGAGGAAGATAAAGATGCCAAAAATATGTTTGATAGGATAGGGAAAAT
AGTGCATGACAAAGTAAAAGAGGAAGCTAAAAAATATATTGAAAAATTGAAAGGAGATTTGA
AGAAAGCAACAAATCGTAGTGGGGAAACAGCATACACCCTTGATCCGTGCATGTTTGATTATA
CTACACATTTTGATGCTAACAGTGAACGGCATCCGTGCAAAAA
TTTAAAAGGAATTACAAATGAAGAACGTTTTTCGGATACACTTGGTGGCCAGTGTACTAAGGAA
AAAATAAGTGGTAGTACAAATACATGTGGTGCTTGTGCTCCATACCGACGCTTACATTTATGTA
ATCATAATTTGGAAACCATAAACAATACAACGTCGATGACGCATAAGTTGTTGGCAGAAGTGT
GTTATGTAGCAAAAGAGGAAGGGGCATCAATAACACGAGATTATCCAAAATATCAAACAACAT
ATGAGGGTTCTGGTTTTACATTGTGTACTATGCTGGCACGAAGTTTTGCAGATATAGGAGACAT
CGTACGCGGTAAAGATCTTTTCCTCGGTAATGATAAAGAAAAAGAAAAAAGAAAACAATTAGA
TGATAAGTTGAAAGATATTTTCAAGCAAATACATAAGGAAGTGACGAAGACGAATAGCGCGTT
ACAAGCTCGCTACGAAGGTGATAAAGAAAATTTTTATCAATTACGAGAAGATTGGTGGGATGC
GAATCGCGCCACAGTATGGAAAGCCATCACATGTAAGGCGGACACTGGTAATGCATACTTTCG
ACCAACGTGCAGTGATGGAAAGAGTCAGTCTCAAGCTAAAAACAAATGCACGTGTAACAATGG
TGATGTCCCCACATATTTTGACTATGTGCCGCAGTTTCTTCGCTGGTTCGAAGAATGGGCAGAA
GACTTTTGTCGATTAAGGAAACATAAATTAAAAGATGCTAAAGAACAATGTCGTAAACCAAAT
GGTGAAGATAAATATTGTGATCTTAATAGGTATGATTGTGAAAAAACAGCTAGCGGAAAACAT
GATTTTTTTGAAGAGGATGTTTGTAAAGATTGTCAGTATTCGTGCGCTCGTTTTGTAAAATGGAT
AGATAACCAAAAAAAAGAATTTGAAAAACAAGAAAAAAAAATATACAAAAGAAATAAAAAAAG
ATCATGGTACAACATTACAAGTTGGAAAAACAACTATTAATAACTTATATGTAGATGATTTTTA
TAAAATACTGAAAAAATATTATCCAACAGTTGATAAATTTTTAGAAAAATTAAGTAAAGAAAA
AATATGCGAAAAGCAGCCTGAAGTGGAAGGAAAAGGAAAGTCTATCGATTTCAATGATGAACC
TGACGACATATTTTCTCGTACAAAATATTGTCGTGCATGTCCTTTGTGTGGAGTAAATGGCCCCA
AAGGAAAATGGAAAGATATAGATGATGGAGTATGTGCAAATTTAAATAAAAAAAAGAATTAC
AAAGAAGATAATATTACCGATATACCAGTACTTACCCCTGAAAAAGGAAAGACAGGTATACTA
AAAAAATATGAAACGTTTTGTGCAACTGGTGTTGGTCAAATTAAAAAATGGGAATGTTATTATG
ATGAAGATAAGCCTAGTGGTCAAAATAATAATTGCATACTAGGAAAATGGGAAAGTTTTACAG
GGGAAGAAGACGTTATGTCCTATAATGCATTTTTTTGGAAGTGGGTTTCAGAAATGTTGGACGA
TTCTATAAAATGGAGGGCAGAACTTGATAAATGCTTAAAAAATGATAAGAAAACATGTGGAAA
AAAAAAATGTAATAGAGACTGTAAATGTTATAAAAAATGGGTTAAAAAAAAAGAAACCGAAT
GGGAAGAAATAGAAAAACATTTTCGCAAGCAAAAAGATATGGAAAATGAAGGATTGAACTTTG
AAATGGCACTTAAAATTCTTTTAAATGATGTTTTTTTGCAAGATATGATAGATGCTAATGGAGA
TCCACAACAAATAGCGAAAATTAAAGAACTGAAGGAAAAGAAAAACGACGAACGTGAGGATT
ATTCCAAAGCGAAAACTATAATTGATTTGTTGATCGATGAAGAAGAACAAGACGCCGATNNNA
ATGTGTAACAAATAATCCAGATAACGAATGCAAACAAGCACCAACAGATGGAGAAAGTCCCTT
GCGTTCCGACACTCCCACACCATCATCACCTGCCCCCTCTTCCCCCCCCAAAAAAGAGGATTCC
GACTCCGAAGAAGAGGAGGAAGAAGAAGAAGGTGAAGGTGAGGATCACACCGCAGAAGACAC

CAAGGTGAACGGGGAGGTGGCACAACCAGAACCAACAACAGATAAGACTACACCACTGGACG
TTTGCAACATAGTGGAAACACTATTTACTACACCGAAAAGCCTCGATGAAGCCTGCAACCAAA
AATATAGCGAACCCAACAGATATTGGGGGTGGAAATGTATACCAACTAGTGGTGGTAGTGGTG
AAGCCAAAAGTCGTCACAAACGTGATCTCGCCCCATCTAGTGGTACTAACCAGGGTTCCATTTG
TGTTCCACCCAGGAGGCGAAAATTATATGTGGGGAAATTGGAACAGTGGGCAAAAACAGTGGT
AGAACCACGGCCGGGTGAGACAACACAGGTTTCCACTTCGGCGGGAACTTCGTCGCCGTCCAA
CTCACGCGACGTCGACTTGCGCAACGCCTTCGTGGAATCGGCCGCGGTTGAGACGTTTTTCTTA
TGGGATAGATATAAAAAAGAGTGGTTGGCACAAAAGAAGGCGGAACAAGCACGACAAAATGG
AGAACTCCTCTTTCTCAACGGCGGATCACCACAACCACCCGGTTCAGGCAGTGATGACAATAAC
CCCCAAAAGAAATTACAAGAAAGTGGTGAAATCCCCCCCGATTTTTTGAGATTAATGTTTTATA
CTTTAGGAGATTATAGGGATATATTATACAGTGGTGATAAGGAAAATGGTAGTAAGTACATGTT
GGTTGAGGATATTAAAAATATATCTGAGAAAATTAAGAGTATTTTGAACAATGAAAATGACGC
CAAAACAACAGCACAACAATGGTGGGATAAAAATGCAAAACATATTTGGAAAGGGATGATTTG
TGCTTTAACCTATGAAGAAAGTGGAGCAATAGGCGGAA
AACCAACAGAAGATCCCACTGTGAAAGAGGCCTTTTTTGGCACCCAAAACGGAAAACCTGGAC
CCCAAAATGGCACAGCCGCAACCCCAACCGGCACATACGAAAAAACCTACAAATACGATAAAG
TGGAACTTAAAGAACAAAATGGTACGGAAGCCAAACCTATCATCGCCTCAGCCTCTAGTGATA
CCCCCACCATCAACAACCCCAAATTGACAGAATTTGTGGTAAGACCCCCCTACTTCCGTTACCT
TGAAGAATGGGGAGAAACATTTTGTCGACAAAGGACACGTATGTTGAAAGATGTGAAGGATAA
TTGTCGTAATAGTGACAATCCAGGTCACCATTATTGTAGTGGGGATGGGCATGATTGTACTGAT
AAAAATCATAGACATAGGAATATGTTTGCAAATTTAGATTGTCGTCCTTGTTATGAACAATGTA
GAAAATATAGAAAATGGATAGATATGAAATTCGTAGAATATCATAAGCAAGAAAAGAAATATA
AAGATGAATATGGAAAATTAACAGAACGTAATTGTAGTGGTAGTGGTGATGATAATAATTGTT
GTGAACAAATAAAAAAACATTCTACTGCTGCAGATTTTTTGAAATCATTGAAACATTGCAAACC
TGGCGACGATGATGGTGATACAGATAATAAACTAGATTTTGAGGAACCTCTCAAAACGTTTGGC
CCTTTAGAATATTGTAAAACATGTCCTCCCAATAAAGTTAATTGTCGTACACGTGGTAGAACTG
ATACAAAACCATGTACACCAGATAATGGAAATAAGTGGCAAAGTGTTTTTGATAAAATACGTA
AAAACAATGAAAATAGTACTACTATTAATGTAGAAATGGTTGATCGTAGGGGAATTAATATTG
ATAAGAAATTTGAACAATTGTTTAAAGAATCATATCTTTTTAAAAGTGTAAGAGAACAAAACTG
GGAATGTAAAGTTATTAATAATGATACGGATGTGTGTCACCTTACTAATTTTAATGACAAAATA
GACCTTAATGACTATACTACATTTAAAGTATTTCTAGAATATTGGTTAGAAGATTTTTTATATGG
TTATTATTTATTGAAAAAAAAAAATCGATCTATGTACACAAAAAGAAGGAAAGACATGTGAGGA
AGATTCTAAAAATGATTGTGCATGTGTGAAAAAAATGGGTAGAACAAAAGAGTACCCAGTGGGG
AAAAATACAAGAACATTTTCAGAAACAAAAAAATGCTAATGGTGATGATATTAAGTCTAAAGT
TAAATTTTTTTTCCAGCAGGAACCGTTTGAAAGTGATCTCAAAAAAGCTAAAGGAAATTTTGAA
AGTTTAGAGGAGTTAGAGACGTCACTTAAATGTAATGACACTGATTCCTCACAAAAGGATATCA
TAGAATGTATTCTTAAAGATCTTCAGAAAAAAATTGACGATTGTAAATCCCAACCTGGCCAAAA
ACAACAACCCTGTGTTGACAACCCCTCCCCTAGTGGTGAAAATTCCATCCTTGACGACGAAAAC
CTTGACCAAACAACGATAGAAATTCCTGGGGTTTGTAATACAGTGGAAGAGCCGGAAGAGACG
ACGAAGGATAAGAAGGAACCAAAAAAGGAACAGGTACCGGAACCTTCACCAAAAAAGAAAGA
TACAATTACATGTAAGACTAATAAATTTAAAAGTACTGAACCTAAAATACCTATGAATTGTGTA
GAAAAAATAGCAAAGGAATTACAAGAGAATTTTGAAAATGATATGGATTGTATATTGAAACAA
AATATAACACCTATTGATTGTACAAATGAGAGTAAACAATCTTCTCCTGGTAATATTAGTTGCG
ACCTGAAAATACCTTTATTGTGCAATAATTATGCATACACAAGAAACCCATGTGATAATAAAGG
AAAAAATCAGTTTAATGTCGACAGGGAATGGAAATGTTATAAAATTTTTAGGCGTCATCGGGA
AAATAAAGGAGTATGTGTACCTCCTAGAAGAGAAATTATGTGCACAAAAGCACTAGAATATAT
GAGAACATCAAATTATTCTAATACTGAAAAATTGTTAAATATAGTATTACTTACAGCAGCATAT
GAAGGAAAAAATTTAAAAGAACAATGGGAAAAAATAAAAGATCGGAAAATAAAAAGGTACGA
ATTATGTGATGCTATGAAATACAGTTTTGCTGATTTAGGTGATATTATAAGAGGAAGAGATAAA
TATACAGACATTAGATATACTGGTAGAAATAGTAAAAAAATTGAGGAAAATTTAAAAAAAGTT
TTTGAAAATATACAAAAAACAAATACATATTTTCAAAAAGAGTATCCCAATCTGAAAACATTTC
GTTCTGCCTGGTGGGATGCTAATAGAGAATATGTTTGGAAAGCAATGACTTGTTCTGCACCAGA
AAATTTCTATTTTGTAAAGAGAGGAAAAGGTGATGGAAGTGATTTCGAAGTTTTAACATTTTCA

GAACATATAAAATGTGGACATAAAGAACCTCCACCTGTTGATGATTATATACCCCAACGTTTAA
GATGGATGACTGAATGGTCTGAATATTTCTGTAAAGCAATGAATAAAAATATTGATGAAATGA
AAGTTGAATGTGATAAATGTACAAGTGGAAAATGTTCAAATGATACTGAGGGAAAATTTTGTA
TCCGGTGTAAAGAAAAATGTAAATCTTATAGTGAATTTATTAATAAATGGAAAGAACAGTTG
GAAACACAATCAAAATCATATAAAAAATTGTATGAAATGTCTGAAACTTATAGTAGTTTTAGTG
AGGATGTTAAATTGTTTGTAAAAAAAATTGAAGGAAAATCAAAAGGATTGTTCAGTTAAAAACG
CATTTGAATATATACATAAAACGAGTAATTGCGTCAATTATAAATTTAATGAAAATGATGGTTC
TTCTAATATACGATCATATGCTTTCGAAGAAACACCAAAAAGTTATAAAGAAGCTTGCAGTTGT
ACATTACATTCTAAGAATCCATTGGATAATTGTCCTACCGATCAAAACAAAGATGGATGTAAGG
AATTACAAACTTGTACCTTCTGCTCGAAGAATGATTATGATAATAATCTTGATAATTGGAACGC
ATACCTTGTTCTTAATAGTTCAGATGATAACAAAGGTGTATTGATTCCTCCAAGAAGAAGACAT
TTATGTACAAGACCTATCACTGCA

>X98HB3var10_HB3var14
CAATACTCCCATAACATACGCAATACGCCACCACCACCGCCCACACGAACCATGCAAGCTCCA
AAATATATGTATGACATGACATAATGTAGTCGCGAACGATAAACAAAAATGGTGACGCTAGGA
GGTGGTGGTGGAAGTAGTGGTGGTGAGGATGATATTGATAAAACAAGTGCCAAACATTTATTG
GATAGCATAGGGAAAATAGTGCACGACCAAGTGAAAACTGAAGCTGAAAAACGTGGTAAAGG
CTTGCAAGGAGATTTGAAAGAAGCAAGATTTCCACATCGCAATGGGTTCGATCATCCCCAACA
GAATCCATGCTTATTTGATCACACTAGAGATACTAATGTTACCAGTGGTGGTGGTAAAGAGAAT
CCTTGTTACGGTAGACAAGGTGTTCGTTTTTCTGATACAAAAGGAGCAAAATGTTATTCTTATG
GAATAAAGGGTAATGATAGTAGC
ATTGGTTTTTGTGCTCCTTATAGAAGGTTACATCTGTGTGTTCAAAATCTGGAACAGATAAAGC
CTGATCAAATTACAAGTACTCATAATTTATTGGTTGATGTATTACTTGCAGCAAAATATGAAGG
ACAATCAATAACACGAGATTATCCAAAATATCAAGCAACATATGGTTATTCTCCTTCTGAAATA
TGTACTATGTTGGCACGCAGTTTTGCAGATATAGGTGATATTATACGAGGAAAAGATCTGTATC
GTCGTGATAGTAGAACAGATAAATTAGAAGAGAATTTAAGAAAAATTTTCGGGATAATATATG
GAAAATTGAAGGATGCACAAAGTCATTACAGTGATACTACGAATTTTTATCAATTAAGGGAAG
ACTGGTGGGAACTTAATAGAGAAACAGTATGGAAAGCTATCACGTGCGAAGCTGGGAGTGGTA
AATATTTTCGACCAACATGTAATGGAGGAAAATCTTCGACTCCTAATAAATGCCGGTGTGACGG
CGCAAATACCGATCCCCCAACCTATTTTGACTACGTGCCGCAGTATCTTCGCTGGTTCGAGGAA
TGGGCAGAAGACTTTTGCAGGAAAAAAAAAAAAAAAAGTAGAAAATTTGGAACAACAATGTCG
TGGAAAAAGTGAGGAGGGTGAACCAAGATATTGTGATCGTAATGGATTTGATTGCGAACGAAC
TAAATATAAAAAAGGTTACTTTGTTATAGATAAAGGTTGCAATACGTGTTCTGTTTGGTGTCGT
CTGTATGAATCTTGGATAGATAACCAAAAAAAAGAATTTCTAAAACAAAAAAAAAAAATGTGAA
AACGAAATATCAGGTAAACCTAGGCAAAAACGGGATGCAGGTAGTAGTGGTAGTAGTAGTGAT
GATAATGGGTATGAAAAAAAATTTTATGACATACTTAAAAATGATGGATATGGAAAAGTTGAT
AAATTTTTAGATTTATTAAGTAAAGAAGAAGTATGCAAAAAGTTTAGTGAAGACGAAGGAAAA
ATTGATTTTACCAAACATGATAATAAAAATAATGATCAGAAAGGAACATTTTATCGTTCGAAAT
ATTGTGAAGAATGTCCTCTATGTGGGGTGAAAAAGAAAAGTAATGGTGGTAGTGGTAATCAAT
GGGAAGAGAAAGACACTAATCAATGCACGAGTGGAAACCTTTATACTATTTCAACTAGTGCCA
ACCCTATTGATATCAACGTCCTTTCCTTTGGTGATAAACGCGAAGATAGAGAAACAAAATTAAA
CAAATTTTGTGCTGAAAAAAATGGTGGTGGTGGTGTTGGTGGTCGTGGTGGTGGTGGTGGTAGT
GGTACGGGTGGTCGTGGTGATAGTGGCACTAGTGATAGTAAGGAACTGTATCAAGAATGGAAA
TGTTATAAAGGTGAAGATGTAGAGATAGATGGTGAGGATGAAGACTATGAACACGATTATCAC
AGTGAGGTAGAAAATGCAGGCGGATTATGTATATTGGAAAACAAAAATAAAACGTCTGATAAT
GACCCTAAGGAATTCCAAAAGACGTTCAATAATTTTTTTTACTTTTGGATAGTACGTTTTTTGAA
CGATTCTATGTATTGGAGAGACAAAATTAATAATTGTATAGAAAAAGCCAAAAAAGGAAAATG
TCAAAATGATTGTGAAAAGCTTTGTGGTTGTTTTAAAGAATGGATTGGAAAAAAAAAAAACCGA
ATGGGGGCAAATCAAAACGCATTTTGACACGCAAAAAGGTTTTGACAAAGCAGATATTAGCAG
TCAATTAGGTTTCAACTTACGTATGACCGCTGATTTTGTTCTTAAAACAGTTTTGAAATTAGATG
AACTTTTCAATAATATTAAATCGGGTTATGGGGATGTAAAGGAATTAAAAGGAATTAATAAAA
TATTGGAAGAGGAAAAACAAAAAAGTCAAGCAGAAGTTGGTGCCGACAATCAAAATAATACC

ACAATTGATAAATTACTAAAACATGAAGGAGAAGAAGCCGGTGAATGCCTAAAAACACACAA
AGAAAAATGCGAAAAAAAAAGCAAAACCAGAAAGTGTCGCCCGCTCCGAAGACAACCAACCAG
CACTACCTGACCCCACCGTCAACCCAGACGAAAACGCCGACGACGACGCCGAAGAAGACGAA
GAAGAAGATGGCAACGAAGTCGAGGAGGACGAACCACACACGCAAGAAACGGAACAGGCAAC
AAAAGATGAGGTGACACCAGAACCAAAAGTGGAAGTAAATCCATGTGAAATAGTAAAGACAC
TATTTAGTAGCGTGGAAAATCTCACAAAGGCATGTGAACAAAAATATGGTTACCCACAACGAC
ATTGGGGGTGGAAATGTATAAGTGACAAAACAGCCACACGTGGTGGTGTTACAGCCATAGGTG
GTGGTGCCGATCCCGCCAAAGCTAGTGGTACTAACCAGGGTTCCATTTGTGTGCCACCCAGGAG
GCGAAAACTATACGTGGGGAAATTACAGGAGTGGGCC
